# Detection of a Mitochondrial Fragmentation and Integrated Stress Response Using the Cell Painting Assay

**DOI:** 10.1021/acs.jmedchem.4c01183

**Published:** 2024-07-17

**Authors:** Soheila Rezaei Adariani, Daya Agne, Sandra Koska, Annina Burhop, Carina Seitz, Jens Warmers, Petra Janning, Malte Metz, Axel Pahl, Sonja Sievers, Herbert Waldmann, Slava Ziegler

**Affiliations:** † Department of Chemical Biology, Max Planck Institute of Molecular Physiology, Otto-Hahn-Strasse 11, 44227 Dortmund, Germany; ‡ Compound Management and Screening Center, Max Planck Institute of Molecular Physiology, Otto-Hahn-Strasse 11, 44227 Dortmund, Germany; § Faculty of Chemistry and Chemical Biology, Technical University Dortmund, Otto-Hahn-Strasse 6, 44227 Dortmund, Germany

## Abstract

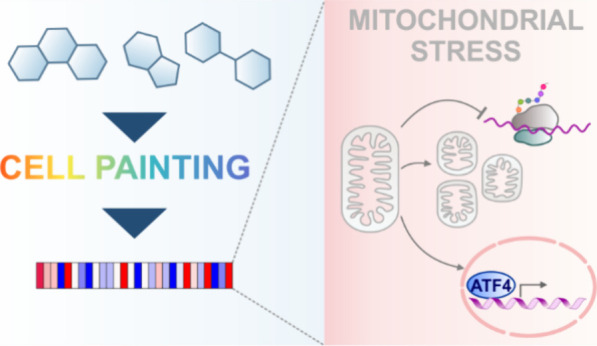

Mitochondria are cellular powerhouses and are crucial for cell function. However, they are vulnerable to internal and external perturbagens that may impair mitochondrial function and eventually lead to cell death. In particular, small molecules may impact mitochondrial function, and therefore, their influence on mitochondrial homeostasis is at best assessed early on in the characterization of biologically active small molecules and drug discovery. We demonstrate that unbiased morphological profiling by means of the cell painting assay (CPA) can detect mitochondrial stress coupled with the induction of an integrated stress response. This activity is common for compounds addressing different targets, is not shared by direct inhibitors of the electron transport chain, and enables prediction of mitochondrial stress induction for small molecules that are profiled using CPA.

## Introduction

Mitochondria are multifunctional signaling organelles that are essential for cellular homeostasis. They are involved in numerous vital processes beyond oxidative phosphorylation (OXPHOS) and ATP production, like lipid oxidation, one-carbon metabolism and pyrimidine biosynthesis, ion uptake, synthesis of Fe/S clusters, anaplerosis, catabolism of various substrates, redox homeostasis, cell signaling, etc.^1^ Mitochondria are linked to aging and diseases such as diabetes type 2, cardiovascular and Alzheimer’s diseases.^2^ Small molecules can selectively modulate mitochondrial targets and processes.^3^ However, compounds may affect mitochondrial function leading to adverse effects^4,5^ Different approaches have been employed to assess impairment of mitochondria such as cell growth studies in the presence of glucose (Glu) or galactose (Gal) (i.e., Glu/Gal assays^6^), metabolic flux measurements or detection of the mitochondrial membrane potential. The characterization of new small-molecule tools and drug candidates would tremendously benefit from the detection of mitochondrial impairment early in the compound discovery process.

Here we report on the use of unbiased morphological profiling by means of the Cell Painting assay (CPA)^7^ for the detection of mitochondrial stress response upon compound treatment in U-2OS cells. In CPA, cells are stained with six different dyes for detection of cell organelles and components (DNA, RNA, mitochondria, Golgi, plasma membrane, endoplasmic reticulum, and actin cytoskeleton).^7,8^ The obtained profiles are then compared with the profiles of reference compounds, i.e., compounds with known targets or modes of action (MoAs) and profile similarity can be used for the generation of target or MoA hypotheses.^9−13^ We identified several small molecules with different targets or mechanisms of action sharing similar morphological profiles, which is induced by impairment of mitochondrial function. This phenotype is detected for some but not all tested iron chelators and is linked to increased levels of mitochondrial superoxide, suppression of mitochondrial respiration after long-term treatment, and activation of cyclic AMP-dependent transcription factor 4 (ATF4) and, therefore, integrated stress response (ISR). Based on a set of similar CPA profiles related to mitochondrial stress, a consensus subprofile for a “MitoStress” compound cluster was extracted according to a recently described approach.^14^ For all CPA-active reference compounds tested by us, biosimilarity to this cluster can easily be assessed via the web app tool https://cpcse.pythonanywhere.com/, which will support the interpretation of results after small-molecule treatment of cells regarding mitochondrial function and may guide the use and prioritization of bioactive small molecules for cell-based studies.

## Results

### Analysis of the CPA Profiles of Ciclopirox

We have screened 4,251 reference compounds and more than 10,000 in-house compounds using CPA.^9−12,14^ U-2OS cells were exposed to the compounds for 20 h, followed by staining of cell compartments and components including the plasma membrane, actin, DNA, RNA, the Golgi, the endoplasmic reticulum, and mitochondria.^7,8^ High-content imaging and analysis resulted in profiles composed of 579 features, which are Z scores representing the differences to the DMSO control.^15^ To describe activity in CPA, we use an induction value (in percent) which is the number of features that are significantly altered compared to the DMSO control, and compounds are considered active for induction ≥5%. Profile similarity (biosimilarity, BioSim, in %) is calculated based on Pearson’s correlation, and profiles are similar if BioSim ≥75% (see the Experimental section for more details). Compounds with biosimilar CPA profiles are expected to share the same target or MoA and can be employed for the generation of target or MoA hypotheses. Our previous analysis of CPA profiles led to the definition of thus far 12 bioactivity clusters^14^ that are based on compound profile similarity, irrespective of different target annotations.^9−12,14^ To simplify the target or MoA prediction using CPA, we recently introduced the concept of subprofile analysis.^14^ For this, features altered in the same direction are extracted from the full profiles recorded for biosimilar compounds that define each cluster. Using the reduced set of features for these compounds, a median profile is generated, termed cluster subprofile, which can be used to calculate cluster biosimilarity.^14^

**Figure 1:**
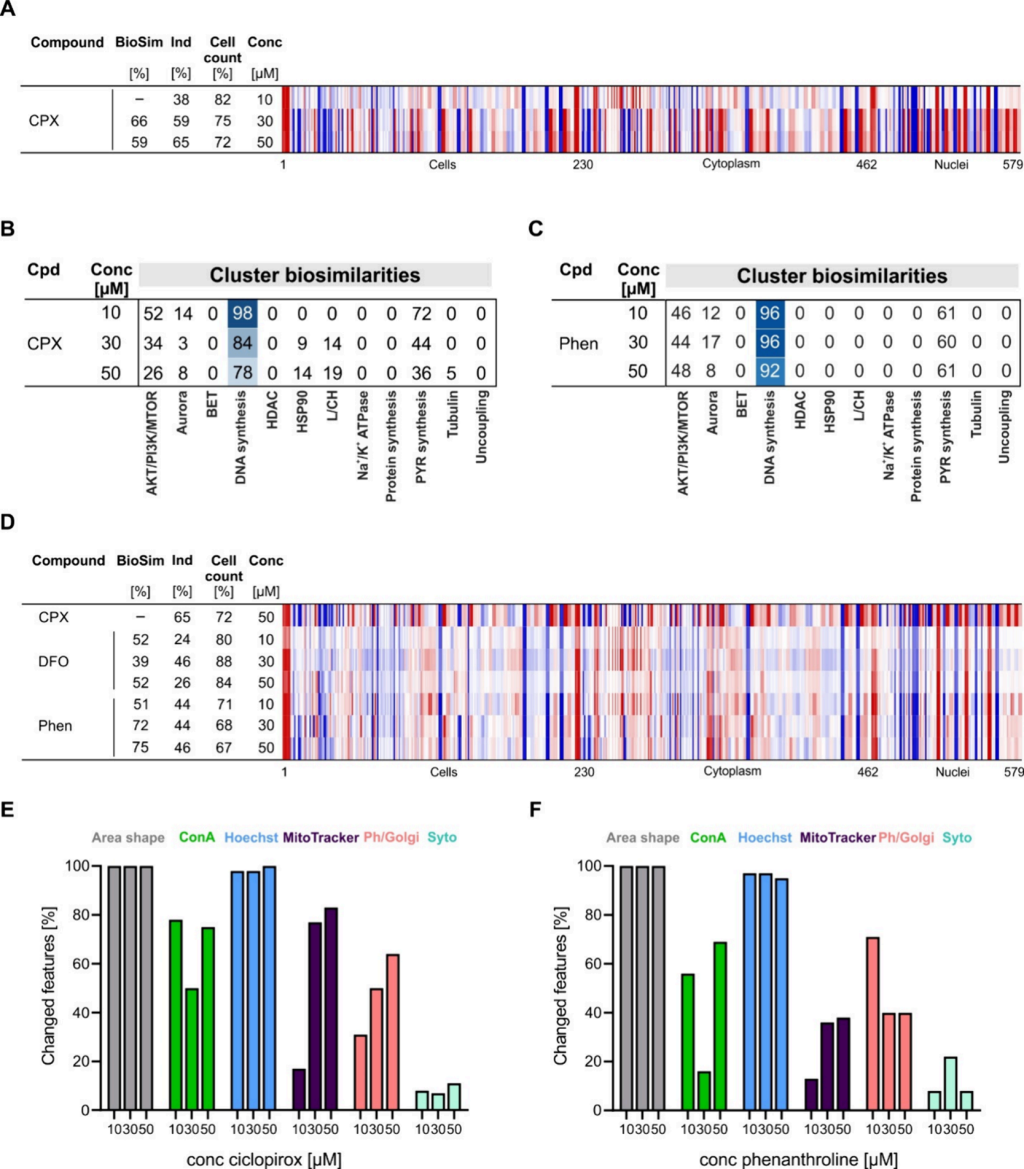
CPA profiles of ciclopirox. (A) Comparison of the profiles of ciclopirox (CPX) at different concentrations. The top line profile is set as a reference profile (100% biological similarity, BioSim) to which the following profiles are compared. Blue color: decreased feature; red color: increased feature. (B) Cluster biosimilarity heatmap for ciclopirox. Percent values are given. (C) Cluster biosimilarity heatmap for the profiles of phenanthroline (Phen). Percent values are given. (D) Comparison of the profiles of DFO, phenanthroline (Phen), and 50 μM ciclopirox. The top line profile is set as a reference profile (100% biological similarity, BioSim) to which the following profiles are compared. Blue color: decreased feature; red color: increased feature. (E, F) Dose-dependent change in dye-related CPA features recorded for ciclopirox (E) or phenanthroline (F). Cpd: compound; BioSim: biosimilarity; Ind: induction; ConA: concanavalin A; Conc: concentration. See also Figure S1.

As recently reported, the CPA profile for the metal ion chelator ciclopirox shares biosimilarity to the profiles of compounds that impair DNA synthesis at a concentration of 10 μM.^10^ However, the profiles of ciclopirox at 30 and 50 μM are not biosimilar to those at 10 μM (Figure 1A). This is in line with a dose-dependent decrease in similarity to the DNA synthesis cluster (Figure 1B). The ciclopirox profiles at 30 and 50 μM were not similar to subprofiles of the remaining 11 clusters pointing toward a thus far unexplored bioactivity in CPA. We compared the profiles of ciclopirox and other iron-chelating agents like deferoxamine (DFO) and phenanthroline.^10^ The profiles of DFO (3–30 μM) and phenanthroline (10 μM) define the DNA synthesis cluster.^14^ The profiles recorded for DFO are biosimilar to each other at all tested concentrations, and similar observations were made for phenanthroline (Figure S1A, B). In line with this, the profiles of DFO and phenanthroline were biosimilar to the DNA synthesis cluster at all tested concentrations (up to 50 μM) (Figures S1C and 1D). No biosimilarity was observed for the profiles of DFO and 50 μM ciclopirox, whereas profile biosimilarity of 75% was detected for 50 μM phenanthroline and 50 μM ciclopirox (Figure 1D). Thus, iron chelators share similar CPA profiles; however, morphological differences are detected at higher concentrations hinting to dose-dependent phenotype shift for some iron chelators.

To gain more information on the type of morphological changes, we compared the altered features in the profiles of ciclopirox at 10, 30, and 50 μM that are related to the individual CPA stains. All Hoechst-related features were changed at the three concentrations, which is related to the impairment of DNA synthesis and the cell cycle (Figure 1E).^10^ However, the number of changed MitoTracker features increased dose-dependently (Figure 1E) pointing toward impairment of mitochondria. To a smaller extent, a similar trend was observed for phalloidin/WGA-related features (Figure 1F). In contrast, treatment with DFO and phenanthroline did not change the MitoTracker-related features in a similar way (Figures 1F and S1D). The altered features with the highest Z-scores for 30 μM ciclopirox were only MitoTracker-related features (see Figure S1E and Table S1). Hence, high concentrations of ciclopirox alter the mitochondrial morphology.

### Similarity to the Profiles of Reference Compounds

**Figure 2:**
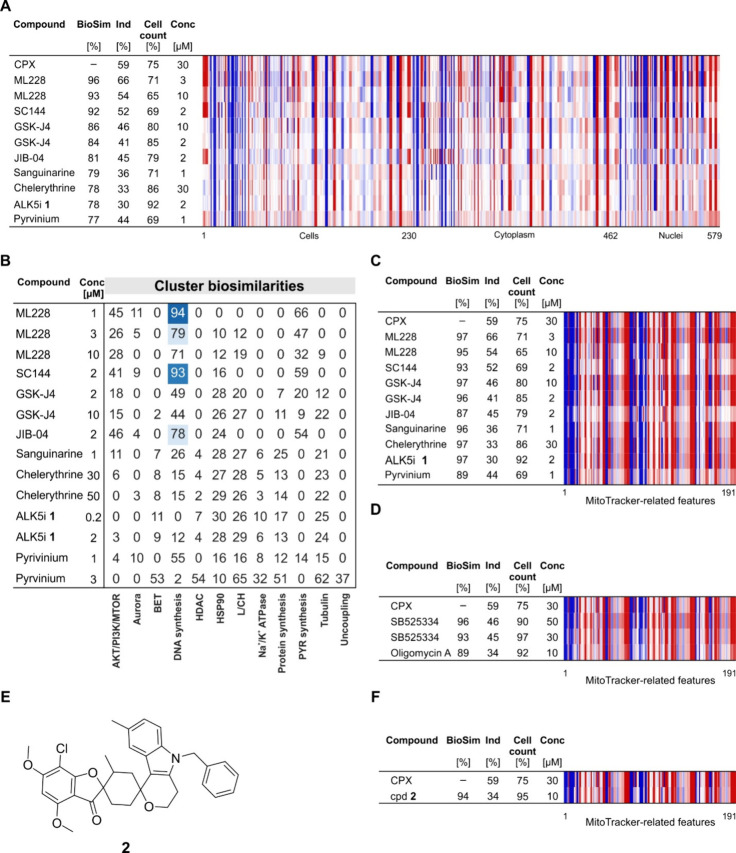
Selected profiles of reference compounds with high biosimilarity to the profile of ciclopirox at 30 μM. (A) Morphological profiles of selected reference compounds with biosimilarity ≥75% to the profile of ciclopirox at 30 μM. (B) Cluster biosimilarity heatmap for the profiles of compounds that are biosimilar to ciclopirox at 30 μM. Percent values are given. (C, D) Biosimilarity of the profiles of reference compounds to the profile of ciclopirox at 30 μM when only MitoTracker-related features are considered. (E) Structure of compound **2**. (F) Biosimilarity of the profile of compound **2** (cpd **2**) to the profile of 30 μM ciclopirox when only MitoTracker-related features are considered. (A, C, D, and F) The top line of the heatmap profile is set as a reference profile (100% biological similarity, BioSim) to which the following profiles are compared. Blue color, decreased feature; red color, increased feature. Cpd: compound; BioSim: biosimilarity; Ind: induction; Conc: concentration; L/CH: Lysosmotropism/cholesterol homeostasis; PYR: pyrimidine (see also Figure S2).

Exploring the profiles of reference compounds that are biosimilar to the ciclopirox profile at 30 μM revealed several biosimilar reference compounds like the Hypoxia Inducible Factor (HIF) pathway activator ML228, the gp130 (IL-6β) inhibitor SC144,^16^ the dual JMJD3/KDM6B and UTX/KDM6A inhibitor GSK-J4, the natural products sanguinarine and chelerythrine, the ALK5 inhibitor **1**(17) and the anthelmintic agent pyrvinium pamoate (Table S2, Figure 2A). These compounds differ in their target annotation (see Table S2). Some of them modulate their targets by chelating metal ions, e.g., ML228 and GSK-J4,^18,19^ and most likely SC144, whose cytotoxicity can be rescued by supplementation of iron, copper, or zinc ions.^16^ Furthermore, diverse activities have been reported for sanguinarine, chelerythrine, and pyrvinium pamoate.^20−22^ Therefore, the profiles of these compounds may result from mixed phenotypes, as already detected for ciclopirox. Indeed, the profiles of ML228 (1 μM) and SC144 (2 μM) display high similarity to the DNA synthesis cluster that is attributed to their iron chelating properties (Figure 2B).^10^

We then compared all of the features related to one of the CPA stains. The biosimilarity of the profiles of these compounds to the ciclopirox profile recorded at 30 μM increased when the MitoTracker-related features were considered, pointing toward an influence of these compounds on mitochondria (see Figures 2C and S2). To focus on only the mitochondrial phenotype, we used all MitoTracker-related features to search for small molecules that share similarity to the profile of ciclopirox at 30 μM and are expected to impair mitochondrial morphology in a similar way. We detected biosimilarity higher than 85% to the MitoTracker-related features for profiles of more than 20 compounds (see Table S3; of note, as only 191 features were compared, we used the more stringent threshold of 85% to judge biosimilarity). The profiles of the compounds displayed again high biosimilarity to the ciclopirox profile at 30 μM (Figure 2C). Moreover, the profiles of the ALK5 inhibitor SB525334 and the F_0_F_1_ ATPase inhibitor oligomycin A were biosimilar to the profile of ciclopirox only when the MitoTracker-related features were compared (Figure 2D).

The F_0_F_1_ ATP synthase is part of the mitochondrial electron transport chain (ETC) and uses the mitochondrial proton gradient to generate ATP. We analyzed the profiles of reference compounds that impair ETC such as the complex I inhibitors rotenone, aumitin,^23^ authipyrin,^24^ and IACS-010759, the complex II inhibitor lonidamine, and the uncoupling agent FCCP. The complex II inhibitor lonidamine was inactive in CPA at 10 and 30 μM. No profile cross-similarity was observed for complex I inhibitors (Figure S3A). Besides targeting complex I, rotenone impairs microtubules,^25,26^ and in CPA, the profile of rotenone is assigned to the tubulin cluster.^9^ The profile of aumitin shows similarity to the L/CH cluster at 10 to 50 μM, whereas the profile authipyrin has only low induction values of 6% at 10 μM. Therefore, the detection of complex I activity in CPA remains elusive. No similarity to the ciclopirox profile was detected for the profiles of inhibitors of ETC and the uncoupling agent FCCP using the full CPA profiles (Figure S3B). As recently reported, the profiles of inhibitors of dihydroorotate dehydrogenase (DHODH, and de novo pyrimidine biosynthesis in general) form a CPA cluster with the profiles of complex III modulators as the activity of DHODH is tightly coupled to complex III.^12^ However, the profile of ciclopirox did not share a similarity with the pyrimidine synthesis cluster subprofile (Figure 1B). Using only MitoTracker-related features, biosimilarity to the ciclopirox profile at 30 μM was detected only for the profile of oligomycin A (Figures 2D and S3C). Hence, the detected phenotype is not related to a direct impairment of the ETC.

### Analysis of Mitochondrial Function

To explore the phenotype induced by 30 μM ciclopirox in more detail, GSK-J4 was selected as a second compound with metal ion-chelating properties as well as SB525334 as the profiles of both compounds do not display biosimilarity to the DNA synthesis cluster (Figures 2B and S4A). In the further analysis, the in-house compound **2** was included as an uncharacterized small molecule since its profile was biosimilar to the ciclopirox profile at 30 μM but did not show any similarity to the 12 clusters (Figures 2E, F and S4B, C).^27^

**Figure 3:**
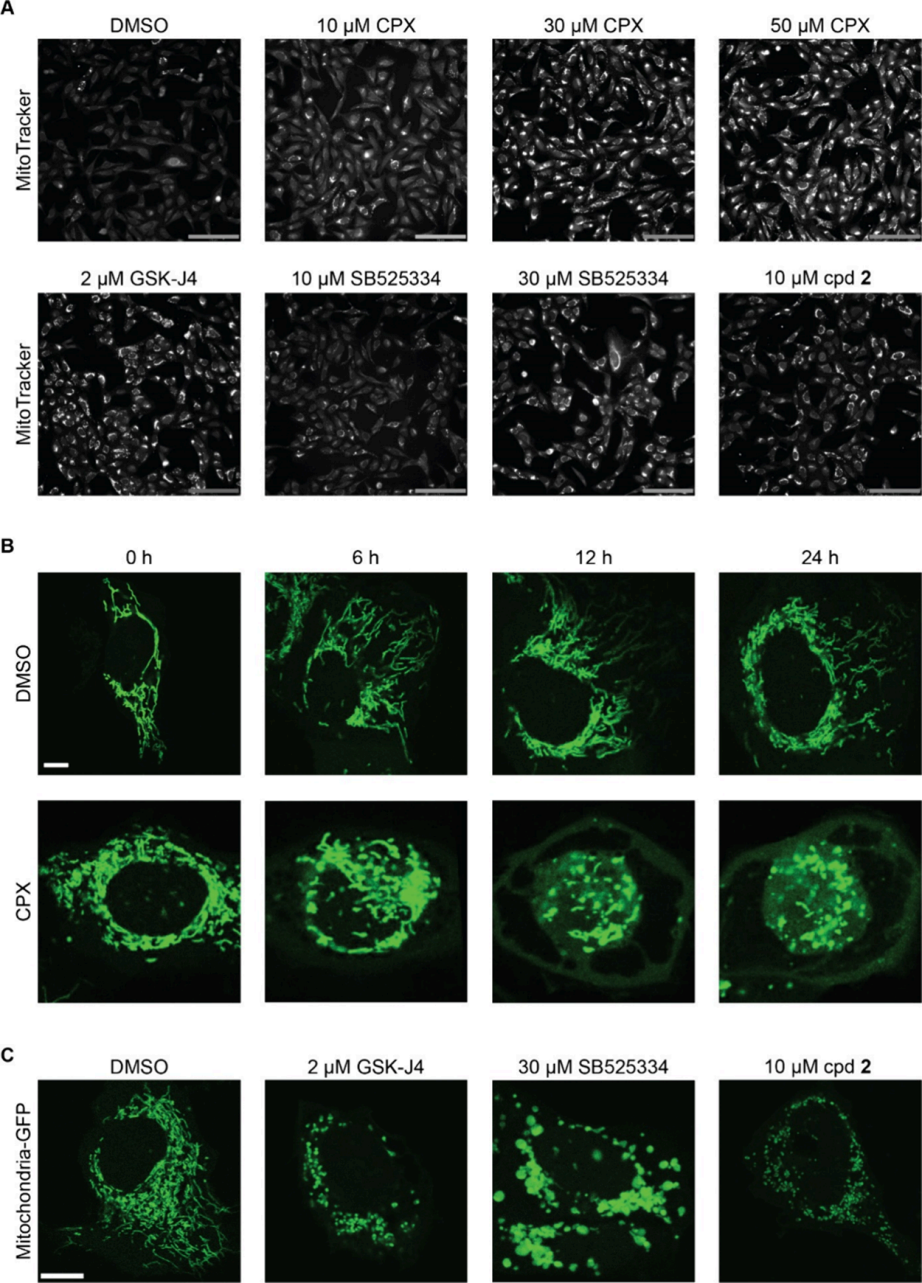
Influence of the compounds on the mitochondrial network. (A) MitoTracker Deep Red staining for ciclopirox (CPX), GSK-J4, SB525334 and compound **2** (cpd **2**). Images from CPA are shown. Scale bar: 176 μm. (B, C) U-2OS cells were transduced with CellLight Mitochondria-GFP BacMam 2.0 and treated with the compounds. (B) Mitochondrial morphology was observed in real-time over 24 h after treatment with 30 μM ciclopirox. Images at selected time points are displayed. Scale bar: 10 μm. (C) Mitochondrial morphology after treatment with GSK-J4, SB525334 and compound **2** for 24 h compound. Scale bar: 10 μm. See also Movies S1, S2, and S3.

Mitochondria are dynamic organelles that undergo highly coordinated processes of fusion and fission and can rapidly change their shape and function according to the physiological needs of the cells.^28,29^ Fusion results in the generation of mitochondria that are interconnected and these are present in metabolically active cells.^30^ Fission results in numerous mitochondrial fragments and mediates removal of damaged mitochondria.^30^ Close inspection of the MitoTracker images revealed altered mitochondrial morphology upon treatment with ciclopirox at 30 and 50 μM with puncta-like staining that may resemble mitochondrial fragmentation (Figure 3A). A similar pattern was observed for GSK-J4, SB525334, and compound **2** (Figure 3B). These changes were dose-dependent as exemplified by the Z scores of features related to granularity, intensity, or contrast of the mitochondrial staining (Figure S4D). To gain insight into the phenotype, the mitochondrial network was monitored for 24 h using CellLight Mitochondria-GFP BacMam 2.0. In DMSO samples, the mitochondria formed elongated structures (see Figure 3C and Movie S1). After treatment with ciclopirox for 10 h, dose-dependent fragmentation of the mitochondrial network became visible, whereas this phenotype evolved faster upon the addition of GSK-J4 (Figure 3A, C, D and Movies S2 and S3). Mitochondrial fragmentation was detected also after treatment with SB525334 and compound **2** (Figure 3A, C).

Mitochondria are involved in the induction of apoptosis and mitochondrial fragmentation occurs early during the apoptotic process^31^ and therefore the detected phenotype may be related to cell death. The influence on cell growth and death was analyzed by real-time live-cell imaging in U-2OS cells over 48 h. Propidium iodide (PI) and caspase 3/7 activity were used as markers of cell toxicity and apoptosis, respectively (Figure S5). Cell growth was hardly impaired by the compounds even after 48 h, and only a slight decrease in cell confluence was detected for 10 μM GSK-J4 (Figure S5). Therefore, the observed mitochondrial phenotype is not related to cell death.

**Figure 4:**
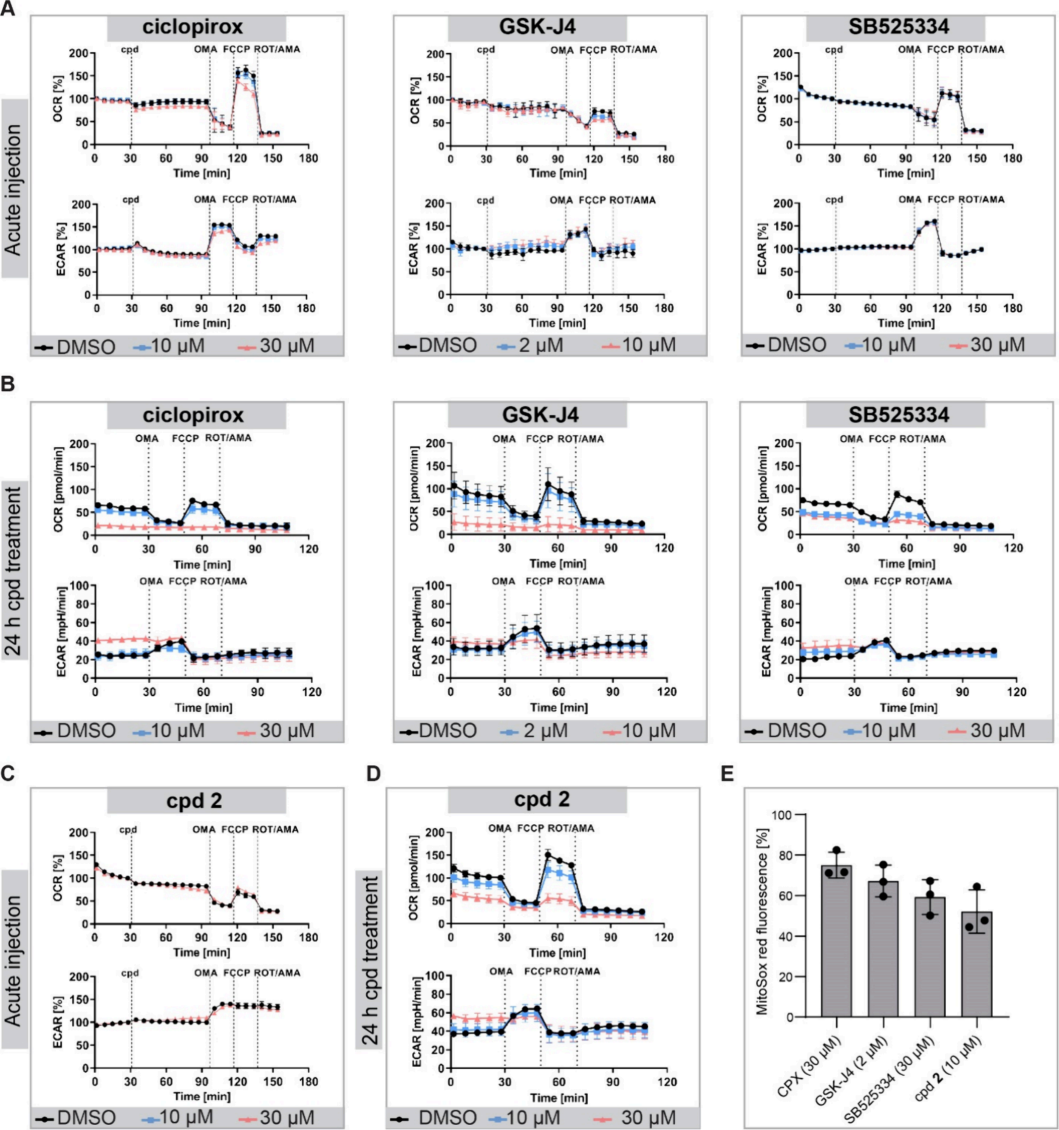
Influence of the compounds on mitochondrial function. (A–C) Influence on mitochondrial respiration as determined using Seahorse technology and a MitoStress Test. The oxygen consumption rate (OCR) and extracellular acidification rate (ECAR) were measured using the Seahorse XF_p_ analyzer. (A and C) Acute injection of ciclopirox, GSK-J4 and SB525334, and compound **2** (cpd **2**) to U-2OS cells. (B and D) U-2OS cells were treated with the compounds for 24 h prior to measuring OCR and ECAR. Mean values are ± SD (*n* = 3). OMA: oligomycin A; ROT: rotenone; AMA: antimycin A. (E) Detection of mitochondrial superoxide using MitoSOX Red after 1 h treatment with the compounds. Data are normalized to CDNB (positive control, set to 100%) and DMSO (negative control, set to 0%). Mean values are ± SD (*n* = 3).

The influence on mitochondrial respiration by means of metabolic flux analyses was assessed using Seahorse technology and determined the oxygen consumption rate (OCR) and extracellular acidification rate (ECAR) as measures of mitochondrial respiration and glycolysis, respectively (Figure 4A–C). Hardly any influence on OCR and ECAR was observed after acute injection of ciclopirox, GSK-J4, SB525334, and compound **2** to U-2OS cells (Figure 4A, C). However, dose-dependent suppression of oxygen production was detected after 24 h of treatment with ciclopirox and GSK-J4, which was almost completely suppressed at 30 μM ciclopirox and 10 μM GSK-J4 (Figure 4B). Similar results were obtained for SB525334 and compound **2** (Figure 4B, D). The drop in OCR may result in an increase in ECAR, which occurs as a compensatory flux.^32^ Indeed, increased extracellular acidification was detected for all compounds at the highest tested concentrations (Figure 4B–D). These findings point toward suppression of mitochondrial respiration by the compounds that evolves over time.

Loss of mitochondrial membrane potential or increased production of reactive oxygen species (ROS) can lead to mitochondrial fragmentation.^28,33^ The influence of the compounds on the mitochondrial membrane potential was assessed using TMRE (tetramethylrhodamine, ethyl ester) as a marker.^34^ TMRE freely crosses membranes regardless of the potential and localizes to active mitochondria due to its negative charge, while it cannot accumulate in depolarized, i.e., dysfunctional mitochondria.^35^ After 24 h incubation of cells with the compounds, no notable loss of membrane potential compared to the controls could be discerned (Figure S6), while FCCP showed a pronounced reduction of TMRE intensity that is in line with its uncoupling activity.^36^ Furthermore, all four compounds increased the level of mitochondrial superoxide as detected with the fluorogenic indicator MitoSox Red^37^ which indicates the induction of oxidative stress (Figure 4E).

### Proteome-Wide Analysis upon Compound Perturbation

**Figure 5:**
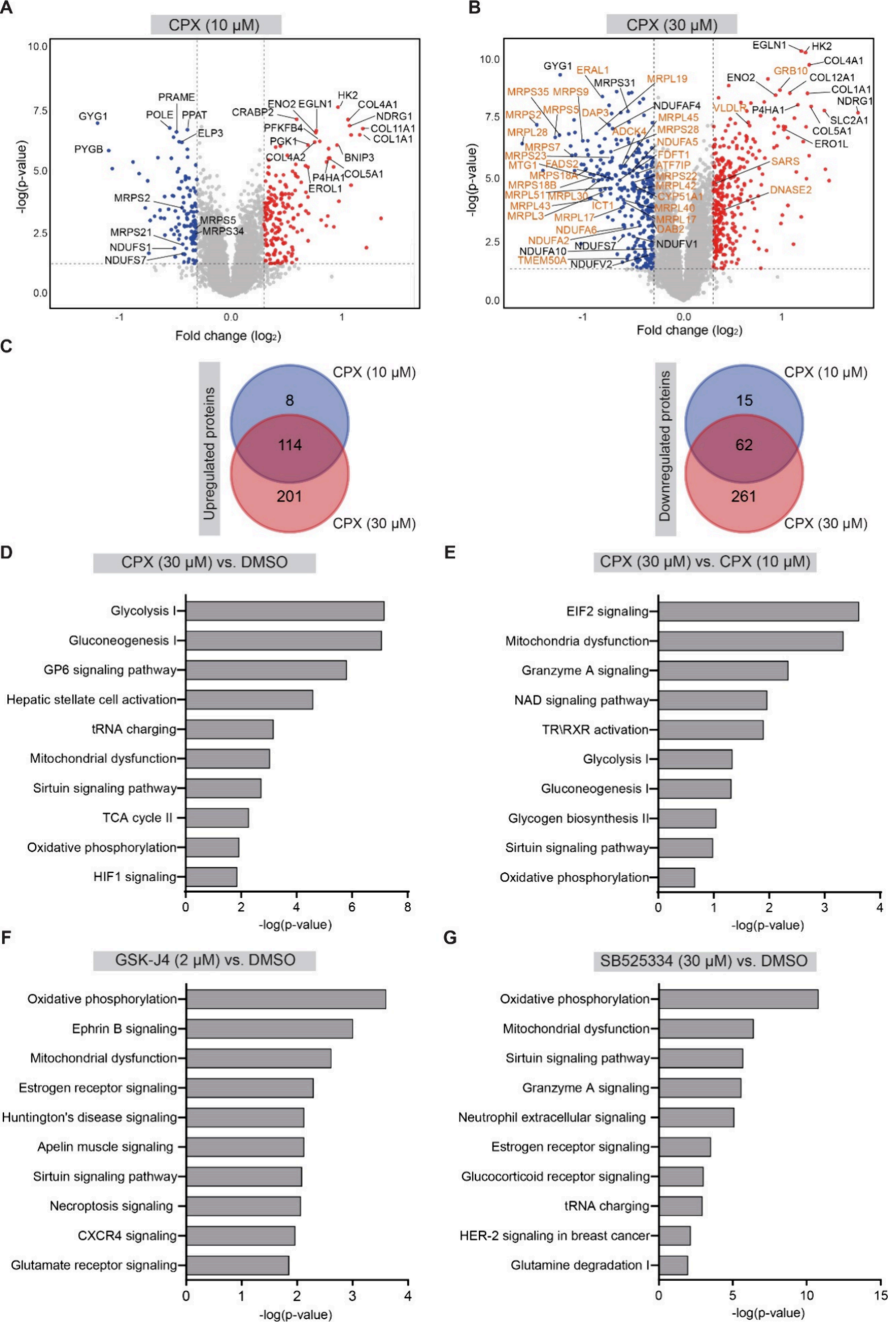
Global proteome profiling and pathway enrichment analysis. (A, B) Volcano plot of log2 fold changes for ciclopirox at 10 and 30 μM upon 24 h compound treatment. Red circles: upregulated proteins; blue circles: downregulated proteins; orange circles: proteins found regulated in Quiros et al. Volcano plots were visualized using VolcaNoseR.^42^ (C) Comparison of the number of upregulated and downregulated proteins by 30 μM vs 10 μM ciclopirox. See also Table S4. (D,E) Pathway over-representation analysis for 30 μM ciclopirox in comparison to DMSO (D) or to 10 μM ciclopirox (E). (F,G) Pathway over-representation analysis for 2 μM GSK-J4 (F) or 30 μM SB525334 (G) compared to the DMSO control. TCA: tricarboxylic acid cycle (see also Figure S7 and Tables S5 and S6).

**Figure 6:**
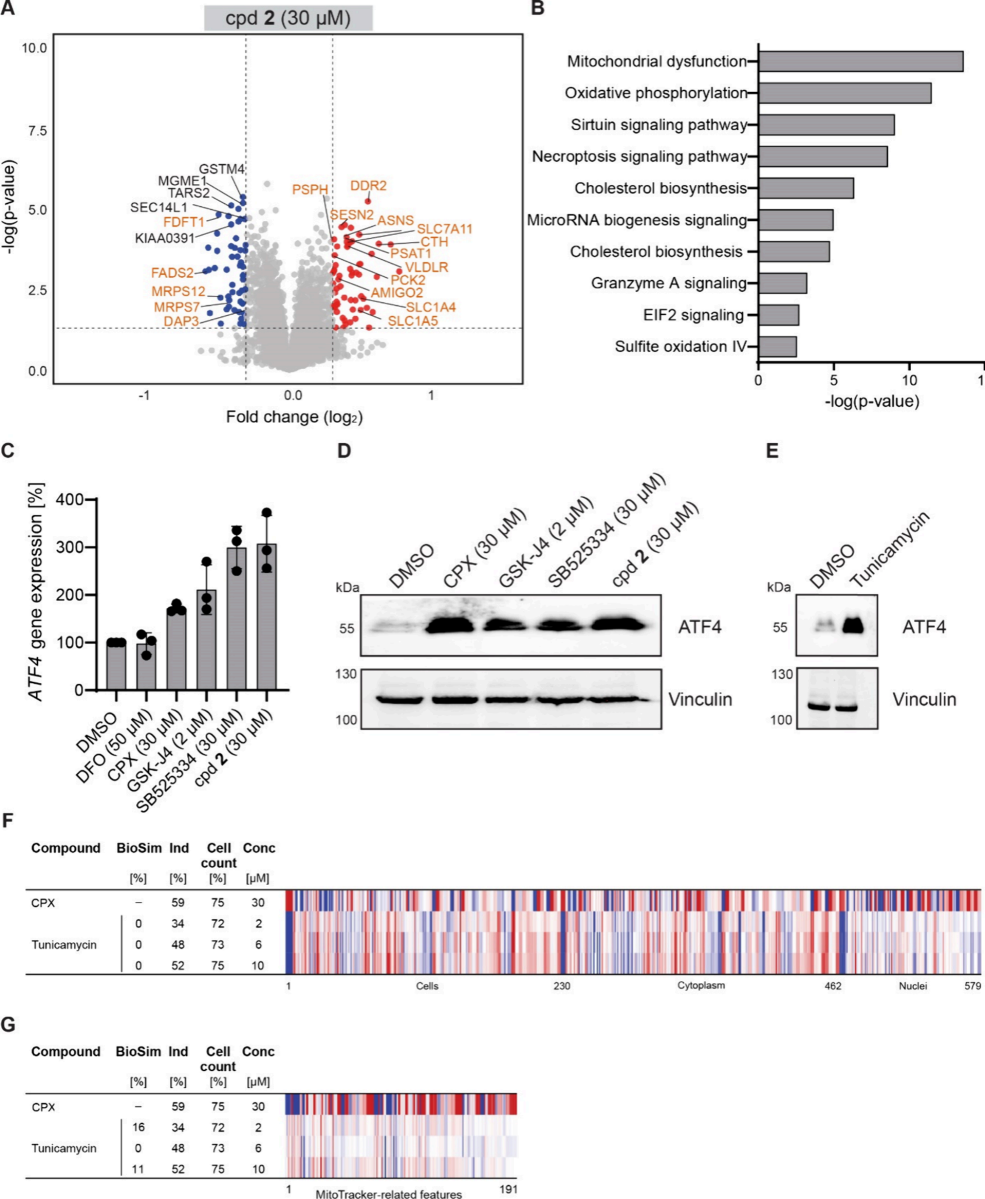
Global proteome profiling for compound **2** and ATF4 regulation. (A) Volcano plot of log2 fold changes for 30 μM compound **2** upon 24 h compound treatment. Red circles: upregulated proteins; blue circles: downregulated proteins. orange circles: proteins found regulated in Quiros et al. Volcano plot was visualized using VolcaNoseR.^42^ (B) Pathway over-representation analysis for compound **2**. See also Table S6. (C) Upregulation of the *ATF4* gene expression upon compound treatment. U-2OS cells were treated with the compounds for 24 h prior to detection of *ATF4* mRNA levels using RT-qPCR. Mean values ± SD (*n* = 3). (D and E) Detection of ATF4 protein levels. U-2OS cells were treated with the compounds for 24 h prior to detection of ATF4 and vinculin as a reference protein using immunoblotting. Tunicamycin: 1 μg/mL. Representative blot is shown (*n* = 3). See Figure S9 for the full blots. (F and G) Comparison of the profiles of tunicamycin to the ciclopirox profile at 30 μM. (F) Full profiles. (G) Comparison of MitoTracker-related features. The top line profile is set as a reference profile (100% biological similarity, BioSim) to which the following profiles are compared. Blue color: decreased feature; red color: increased feature. Cpd: compound; BioSim: biosimilarity; Ind: induction; Conc: concentration.

To gain insight into the modulated pathways, we explored the difference in the proteomes upon treatment of U-2OS cells for 24 h with ciclopirox, GSK-J4, SB-525334, and compound **2** (see Figures 5, 6, and S7). In the presence of ciclopirox, more than 400 proteins were differentially regulated at 30 μM as compared to ca. 200 regulated proteins at 10 μM ciclopirox (see Figure 5A–C and Table S4). 114 proteins were upregulated, and 62 proteins were downregulated at both concentrations (Figure 5C and Tables S4 and S5). Pathway over-representation analysis of the proteome at 30 μM ciclopirox linked the altered levels of these proteins to modulation of glucose metabolism, glycolysis, oxidative phosphorylation (OXPHOS), mitochondrial dysfunction, and hypoxia-inducible factor 1 (HIF1) signaling (Figure 5D).

Glucose metabolism and HIF1 signaling are linked since during hypoxia, cells switch their metabolism from mitochondrial respiration to glycolysis to meet the bioenergetic requirements.^38^ Ciclopirox upregulates several proteins such as the HIF1 prolyl hydroxylase Egl nine homologue 1 (EGLN1), hexokinase-2 (HK2), heme oxygenase 1 (HMOX1), and the glucose transporter solute carrier family 2 member 1 (SLC2A1, also known as GLUT-1) (Tables S4–S6). These proteins are involved in HIF1 signaling, or their expression is induced by the HIF1 transcription factor. The HIF1-α protein is regulated on the protein level, as prolyl hydroxylation of HIF1-α by HIF prolyl hydrohylase (HIF PHD) leads to its proteasomal degradation via the E3 ligase Von Hippel-Lindau (VHL). As HIF PHD requires oxygen for its enzymatic activity, HIF1-α levels are low during normoxia and increase during hypoxia. Furthermore, HIF PHD requires Fe(II) as a cofactor. Therefore, iron chelators induce HIF1 response by interfering with the activity of prolyl hydroxylases, thereby stabilizing HIF1.^39^ Ciclopirox has been reported to stabilize HIF1-α under normoxic conditions.^40,41^ We therefore explored whether compounds biosimilar to 30 μM ciclopirox also regulate HIF1 levels. No modulation of HIF1 signaling was observed in the proteome profiling for GSK-J4, SB525334, and compound **2** (Figures 5F, G, S7, and 6A, B). Moreover, the compounds neither induced HIF1-α -dependent reporter expression nor increased HIF1 protein levels (Figure S8A, B). In line with this, only ciclopirox scored as a direct iron chelator using a ferrozine-based iron chelation assay (Figure S8C). Hence, the modulation of HIF1 signaling is not the common denominator for the detected phenotype in CPA.

Comparison of the proteomes at 30 and 10 μM ciclopirox revealed eukaryotic translation initiation factor 2 (EIF2) signaling as the most significantly modulated process at 30 μM ciclopirox (Figure 5E). Among the downregulated proteins upon treatment with 30 μM ciclopirox vs 10 μM ciclopirox were several mitochondrial ribosomal proteins (MRPs) and NADH-ubiquinone oxidoreductase subunits (NDUFs), which are complex I components (Figure 5A, B, Table S4). The most significantly regulated pathways for all tested compounds were oxidative phosphorylation and mitochondrial dysfunction (Figures 5F, G and 6A, B). Modulation of MRPs and/or NDUFs by small molecules has been observed for small molecules that induce mitochondrial stress response such as FCCP, doxycycline, actinonin, and MitoBLoCK-6 and these changes were only mapped on proteome but not transcriptome level.^43^ In parallel, these compounds induced the expression of genes that are regulated by cAMP-dependent transcription factor 4 (also known as activating transcription factor, ATF4).^43^ ATF4 expression is repressed under normal conditions and induction of ATF4 is a hallmark of the integrated stress response (ISR).^44^ ISR is a signaling pathway that is activated as a response to altered physiological conditions such as endoplasmic stress, hypoxia, glucose or amino acid deprivation, or viral infections, which all lead to phosphorylation of EIF2α.^45^ As a consequence, global protein translation is reduced, while ATF4 expression helps the cell to recover and survive.^45^ The extent and duration of ISR determine cell fate and may also lead to cell death.

Several proteins that were found downregulated by Quiros et al. were also downregulated by ciclopirox, SB525334, and compound **2** but not by GSK-J4. Proteins regulated by 30 μM ciclopirox displayed the highest overlap (see Table S7). Moreover, some of the genes or proteins that were reported as upregulated by Quiros et al. were present also at higher levels upon treatment with ciclopirox, GSK-J4 and compound **2**, and compound **2** showed the highest overlap (Table S8). These findings suggested that the compounds may induce ATF4 transcriptional response and ISR. Therefore, we explored whether the compounds affect ATF4 expression. Whereas no ATF4 mRNA and protein was detected in the control condition, ciclopirox, GSK-J4, SB525334, and compound **2** increased *ATF4* gene expression (Figure 6C). In line with this result, the ATF4 protein was detected upon treatment with all four compounds (Figure 6D), indicating the activation of ISR. In contrast, iron chelator DFO, whose CPA profiles are not biosimilar to the profile of 30 μM ciclopirox, does not stimulate *ATF4* expression (Figure 6C). Tunicamycin is a natural product that decreases N-glycosylation in cells and induces endoplasmic stress and ATF4 expression.^46^ We detected increased levels of the ATF4 protein upon treatment of U-2OS cells with tunicamycin (Figure 6E). However, the CPA profiles of tunicamycin were not biosimilar to the profile of 30 μM ciclopirox, using both the full profiles and only MitoTracker-related features (Figure 6F, G). Hence, CPA can distinguish between endoplasmic and mitochondrial stress/ISR, even though both operate via induction of ATF4. These findings reveal the induction of mitochondrial fragmentation and ISR as the common MoA for compounds sharing the same morphological changes as ciclopirox at 30 μM.

### Definition of a Cluster Related to Mitochondrial Stress

**Figure 7:**
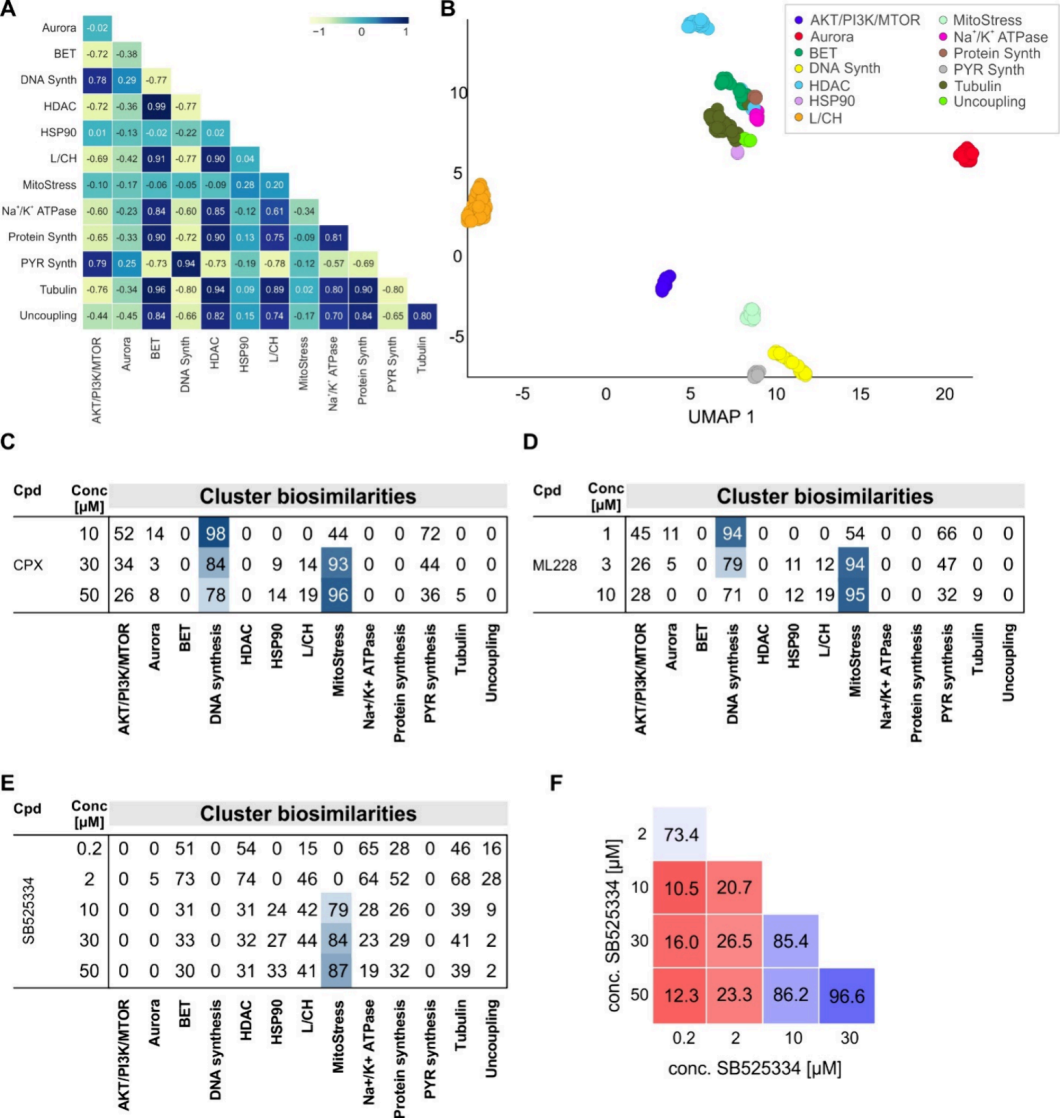
Definition of the MitoStress cluster. (A) Cluster subprofile cross-correlation using Pearson correlation. (B) UMAP plot using the full profiles of the reference compounds that were used to define the bioactivity clusters. Not normalized data, 10 neighbors. (C–E) Cluster biosimilarity heatmap for the profiles of ciclopirox (CPX) (C), ML228 (D) and SB525334 (E). Profile cross-correlation for SB525334. Values are biosimilarity in %. L/CH: Lysosomotropism/cholesterol homeostasis; PYR: pyrimidine. Synth: synthesis. (F) Profile cross-similarity for SB525334.

To enable fast prediction of the observed biological activity, we used the CPA profiles of selected compounds that are biosimilar to the profile recorded in the presence of 30 μM ciclopirox (see Table S9) to extract the characteristic CPA features for this cluster (termed MitoStress cluster) and to obtain a cluster subprofile according to the recently described procedure.^14^ The MitoStress cluster subprofile consists of 294 features and is very different from the 12 defined clusters thus far as demonstrated by the cluster profile cross-correlation and the lower dimension UMAP plot (Figures 7A, B and S10). Thus, the MitoStress cluster is a very valuable new cluster for the analysis of morphological profiling by means of CPA.

A cluster biosimilarity map clearly depicts the morphological phenotypes caused by ciclopirox: whereas at 10 μM profile similarity is observed only to the DNA synthesis cluster, additional similarity to the MitoStress cluster is detected for the ciclopirox profile at 30 μM, while the similarity to the DNA synthesis cluster decreases (Figure 7C). These morphological changes are dose-dependent and are more pronounced at 50 μM (Figure 7C). A similar “cluster shift” was observed for the profile of the iron chelator ML228 (Figure 7D) but not for the profiles of DFO, phenanthroline, deferasirox, and PAC-1 (Figure S11A–D). Hence, all tested iron chelating compounds influence DNA synthesis but not all of them impair mitochondrial function in the tested concentration range. High similarity to the MitoStress cluster is detected for the profiles of further cluster members (see Figure S11E). For the ALK5 inhibitor SB525334, the MitoStress phenotype starts evolving already at 10 μM, and the MitoStress cluster similarity increases in a concentration-dependent manner (Figure 7E). Importantly, for several compounds, the MitoStress phenotype is detected at higher concentrations as exemplified by ciclopirox, ML228, SB525334, and Sal003 (Figures 7C–E and S11F). A cluster shift may result in less biosimilar or even dissimilar profiles at different concentrations of a given compound, as detected for SB525334 and Sal003 (see Figures 7F and S11G). The biosimilarity to the MitoStress cluster for all CPA-active reference compounds, which we have profiled thus far, can be assessed using the web app tool https://cpcse.pythonanywhere.com/.

## Discussion

Detailed knowledge about the targets and processes impaired by small molecules is essential for their proper use as research tools and, more importantly, as drug candidates. Small molecules are usually identified in assays monitoring the modulation of a target or a process, either in vitro, in cells, or in vivo. Thorough characterization of bioactive compounds informs about efficacy and selectivity, and in addition, safety-panel profiling can uncover off-target liabilities. Off-and-on-target adverse effects are best identified early in the compound development workflow, and profiling approaches in cells such as transcriptomics, proteomics, and morphological profiling provide an unbiased view of various bioactivities of small molecules. CPA determines morphological profiles upon perturbation^13^ and, in principle, comparison to the profiles of annotated compounds should lead to target hypotheses. In fact, often similarity in CPA profiles is not linked to the same target annotation, which may result from off-target activity or impairment of the same pathway but at a different level.^9−12^

Analyzing the CPA profiles of reference compounds revealed a new CPA bioactivity cluster that impairs mitochondrial morphology by inducing a fragmented phenotype and mitochondrial stress. These morphological changes were detected for some, but not all, iron-chelating compounds. Whereas ciclopirox, ML228, GSK-J4, and SC144 induced mitochondrial stress at the tested concentrations, DFO, phenanthroline, deferasirox, and PAC-1 did not. Induction of HIF1 by the chelator desferrioxamine causes mitochondrial fission.^47^ However, while all tested iron chelators inhibit DNA synthesis, they do not share mitochondrial fission and stress as a common MoA. The detected differences for iron chelators underscore the power of morphological profiling in mapping various bioactivities for given compounds, which assists the selection and proper use of these small molecules in cellular studies. Of note, Wheeler et al. identified ciclopirox, ML228, GSK-J4, JIB-04, SC144, and NSC319726 as hits in a screen for macrofilaricidal activity in *C. elegans*(48) and the activity could not be linked to the annotated targets (e.g., histone demethylases, HIF-1α). The macrofilaricidal activity may be due to iron chelation. Alternative MoA is suggested by our CPA study and may link mitochondrial stress to macrofilaricidal activity.

CPA has been employed to study cell and mitochondrial toxicity.^49−53^ By using CPA profiles, gene expression signatures, chemical structural information, and mitochondrial toxicity data, Seal et al. could distinguish between mitochondrial toxicants and nontoxicants based on the morphological profiles.^51^ None of the CPA profiles of the mitotoxic compounds investigated by Seal et al. display similarity to the MitoStress cluster (Figure S12A) and, therefore, impair mitochondrial homeostasis by a different MoA. Trapotsi et al. explored protein-targeting chimeras in CPA along with mitotoxic compounds to train a model for mitotoxicity.^52^ The CPA profiles for the disclosed compounds are not biosimilar to those of the MitoStress cluster (Figure S12B). In line with this, we also did not detect similarity to the MitoStress cluster for compounds directly impairing the mitochondrial ETC. Moreover, the MitoStress phenotype, which we detect after treatment for 20 h, is not linked to toxicity, as we failed to detect cell death even 48 h after compound addition. Hence, we identified a bioactivity cluster linked to mitochondrial fragmentation and stress that can be mapped by using morphological profiling.

The compounds in this cluster cause profound changes in the mitochondrial network architecture that are reminiscent of mitochondrial fragmentation. Several compounds have been reported as fission inducers.^54^ The iron chelator phenanthroline induces mitochondrial fragmentation at 50 μM^55^ and also we detect biosimilarity between the profiles of 30 μM ciclopirox and 50 μM phenanthroline. However, the similarity of the profile of phenanthroline at 50 μM to the MitoStress cluster of 62% is lower than the suggested biosimilarity threshold of 80% for subprofile comparison. In general, the similarity to the MitoStress cluster is detected at higher concentrations, whereas the phenotype differs at lower concentrations, as observed for the iron chelators ciclopirox and ML228 but also for SB525334 and Sal003. Therefore, the morphological profiles for this type of compounds can guide the selection of an appropriate concentration for cellular experiments in order to study the desired mechanism of action without causing mitochondrial stress. This is particularly important considering that the MitoStress phenotype occurs at nontoxic concentrations and may be therefore easily overlooked.

Mitochondrial fragmentation is linked to oxidative stress^28,56^ and we detected increased levels of mitochondrial superoxide by ciclopirox, GSK-J4, SB525334, and compound **2**. The compounds did not attenuate the mitochondrial membrane potential and suppressed mitochondrial respiration only after long-term treatment. ETC dysfunction is linked to mitochondrial fission.^57^ Mitochondrial fragmentation has been reported for ETC inhibitors and uncoupling agents, but for them, we did not detect the fission phenotype in U-2OS cells and biosimilarity to the MitoStress cluster at the tested concentrations after treatment for 20 h. Hence, CPA can differentiate between different types of mitochondrial modulators such as complex III inhibitors (which are assigned to the pyrimidine synthesis cluster), uncoupling agents, and compounds inducing mitochondrial stress.

Besides the four small molecules explored here, the profiles of further reference compounds displayed biosimilarity to the profile recorded for ciclopirox at 30 μM and the MitoStress cluster. Some of them are lipophilic cationic molecules that have been reported to influence mitochondria and may accumulate in these organelles.^58^ For example, the alkaloids sanguinarine and chelerythrine increase ROS levels and attenuate mitochondrial membrane potential.^59^ Pyrvinium pamoate inhibits mitochondrial respiration after 24 h and induces ISR in MOLM13 cells.^60^ The phosphatase inhibitor Sal003 is an inhibitor of the EIF2α phosphatase and thereby promotes EIF2 phosphorylation that ultimately increases ATF4 levels, thus protecting cells from endoplasmic reticulum stress.^61,62^ We detected a similarity of Sal003 to the MitoStress cluster at 10 μM but not at 3 or 6 μM. For its less potent derivative salubrinal, neither similarity to the MitoStress cluster is detected nor profile similarity to Sal003 up to concentrations of 50 μM (Figure S13). The low induction values for salubrinal in comparison to Sal003 (see Figure S13B, C) are in line with its lower potency and explain the observed differences in CPA.

Treatment with 30 μM ciclopirox reduced the levels of numerous MRP and NDUF proteins, which indicates a downregulation of mitochondrial translation and complex I activity. Quiros et al. observed lower levels for several MRPs and NDUFs in a study using mitochondrial stressors such as doxycycline, FCCP, actinonin, and MitoBlock 6.^43^ This regulation occurs on the translation level as no changes in the expression of the corresponding coding genes were detected. An increase in ROS production can cause global inhibition of protein synthesis by different mechanisms and lead to phosphorylation and inactivation of EIF2.^63,64^ At the same time, mRNAs with upstream open reading frame (uORF) are selectively translated such as for the transcription factor ATF4.^65^ ATF4 activates the transcription of genes involved in amino acid transport, serine biosynthesis, one-carbon metabolism, antioxidant defense and proteostasis,^57,59^ a stress pathway known as an integrated stress response (ISR). In line with this, Quiros et al. observed the upregulation of genes involved in serine biosynthesis and one-carbon metabolism and demonstrated the activation of ATF4 and ISR.^43^ Similarly, we detected elevated levels of *ATF4* mRNA and ATF4 protein in the four studied compounds, indicating that the observed CPA phenotype is due to oxidative stress, mitochondrial fragmentation, and ISR. This is supported by the fact that the iron chelator DFO fails to induce mitochondrial fragmentation and ATF4 expression and, therefore, this phenotype is not caused by general iron chelation.

Mitochondrial stress duration has a different impact on ATF4 levels: whereas ATF4 is induced after short-term stress, ATF4 signaling is attenuated after long-term stress to allow for protection against long-lasting inhibition of protein synthesis.^64^ This finding together with the type and potency of the mitochondrial stressor may explain why some known mitochondrial stressors such as FCCP or antimycin A lead to a different CPA phenotype: FCCP shares a distinct phenotype with other, structurally dissimilar uncouplers, whereas the profile of antimycin A is assigned to the pyrimidine synthesis cluster.^12,14^

The extent and duration of ISR determine the cell fate and may lead to cell death. Several compounds share in CPA the mitochondrial fragmentation/ISR phenotype. Considering that ISR is implicated in different diseases,^66^ strategies for pharmacological modulation of ISR have been explored.^45,66^ ISR induction by the small molecule BTM-3566 was shown to cause growth arrest and apoptosis in diffuse large B-cell lymphoma (DLBL) and complete regression in patient-derived xenografts,^67^ demonstrating that induction of ATF4 response may be beneficial for cancer treatment. On the other hand, ISR has been linked to an increase in ponatinib-induced cardiotoxicity^68^ and drug-induced liver injury.^69^ In these studies, ISR was detected upon transcriptome or proteome profiling. Cell painting provides an alternative approach to rapidly predict the induction of mitochondrial stress/ISR. To allow for easy detection of compounds inducing this phenotype, we defined the MitoStress cluster by extracting a median profile of the observed morphological changes using a recently described procedure.^14^ Biosimilarity to this cluster subprofile would suggest the induction of mitochondrial stress and ISR. For all CPA-active reference compounds that we have screened thus far, biosimilarity to the MitoStress cluster subprofile can be queried using the web app tool https://cpcse.pythonanywhere.com/. Of note, the CP profile for ponatinib does not show similarity to the MitoStress cluster. Instead, we detect high cluster similarity to the lysosomotropism/cholesterol homeostasis cluster (see Figure S14). This activity is attributed to the physicochemical properties of the compound, i.e., it is a lipophilic and weakly basic compound.^11^ However, the subprofiles of ponatinib and SB525334 containing only the MitoTracker-related features of the MitoStress phenotype were biosimilar, demonstrating that the MitoStress phenotype can also be resolved for lysosomotropic compounds.

## Conclusions

Using CPA, we identified a morphological phenotype that is related to mitochondrial stress and is linked to the activation of ATF4 and the integrated stress response. This MoA is shared by compounds with different targets that indirectly suppress mitochondrial respiration and increase ROS levels in mitochondria and differs from the CPA profiles of direct inhibitors of ETC or uncouplers. The newly defined MitoStress cluster and cluster subprofile will enable the rapid prediction of this MoA for compounds profiled using CPA.

## Experimental Section

### Materials

#### Chemicals, Reagents and Kits

CellEvent caspase-3/7 green (Thermo Fisher Scientific; Cat# C10427); 1-chlor-2,4-dinitrobenzol (CDNB) (Sigma-Aldrich; Cat# 237329); CellLight mitochondria-GFP, BacMam 2.0 (Thermo Fisher Scientific; Cat# C10596); cell mito stress test kit (Agilent; Cat# 103010-100); DC protein assay kit II (Bio-Rad; Cat# 000112); DMEM medium (high glucose) (PAN Biotech; Cat# P04-03550); DNase-free RNase A (Thermo Fisher Scientific; Cat# EN0531w); dual-luciferase reporter assay system (Promega; Cat# E1960); ferrozine (Thermo Fisher Scientific; Cat# 10522194); fetal bovine serum (Gibco; Cat# 10500-084); Hoechst 33342 (Cell signaling; Cat #4082S); iron(II) sulfate heptahydrate (Sigma-Aldrich; Cat# F8633); lipofectamine 2000 (Thermo Fisher Scientific; Cat# 11668030); Mito Tracker deep red (Thermo Fisher Scientific; Cat# M22426); MitoSOX red dye (Thermo Fisher Scientific; Cat# M36008); MycoAlert mycoplasma detection kit (Lonza; Cat# LT07-318); nonessential amino acids (PAN Biotech; Cat# P08-32100); propidium iodide (Sigma-Aldrich; Cat# P4864); Quanti Tect reverse transcription kit (Qiagen; Cat# 205313); Qubit RNA BR assay kit (Thermo Fisher scientific; Cat# Q10210); Seahorse XF Calibrate (Agilent; Cat# 100840-000); Seahorse XF DMEM medium pH 7.4 (Agilent; Cat# 103575-100); Seahorse XFp mito stress test kit (Agilent; Cat# 103010–100); sodium pyruvate (PAN Biotech; Cat# P04–43100); Sso Advanced Universal SYBR Green Supermix (Bio-Rad; Cat# 1725274); and tetramethylrhodamine ethyl ester (TMRE) (Thermo Fisher Scientific; Cat# T669) were purchased and used.

#### Antibodies

HRP (Goat anti-Rabbit IgG) (Thermo Fisher Scientific; Cat# 31460); IRDye 680RD (donkey antimouse) (LI-COR Biosciences; Cat# 26-68072); IRDye 800CW (donkey antirabbit) (LI-COR Biosciences; Cat# 926-32213); IRDye 800CW (goat antimouse) (LI-COR Biosciences; Cat# 926-32210); Mouse monoclonal anti-HIF1-α (Novus; Cat# NB100-105); Rabbit monoclonal anti-ATF4 (Cell Signaling; Cat# 11815).

#### Cell Lines

HEK293T cells (human embryonic kidney cells) (ATCC; Cat# CRL1268) and human U-2OS cells (CLS; Cat# 300364) were used.

### Cell Lines

The U-2OS female human bone osteosarcoma cell line was cultured in Dulbecco’s Modified Eagle’s medium (DMEM, high glucose) supplemented with 4 mM l-glutamine, 10% fetal bovine serum, 1 mM sodium pyruvate, and nonessential amino acids. The cells were incubated at 37 °C and 5% CO_2_ in a humidified atmosphere. The MycoAltert Mycoplasma Detection Kit was used monthly according to the manufacturer’s instructions to detect contamination with mycoplasma. Cells were always tested free of mycoplasma.

### Cell Painting Assay

The Cell Painting assay follows closely the method described by Bray et al.^8^ as recently reported.^14^ “Initially, 5 μL U-2OS medium was added to each well of a 384-well plate (PerkinElmer CellCarrier-384 Ultra). Subsequently, U-2OS cells were seeded with a density of 1600 cells per well in a 20 μL medium. The plate was incubated for 10 min at the ambient temperature, followed by an additional 4 h incubation (37 °C, 5% CO_2_). Compound treatment was performed with an Echo 520 acoustic dispenser (Labcyte). Different concentrations of DMSO were used as controls dependent on the used compound concentration; e.g., 0.1% DMSO was used as a control for the profiling of compounds at 10 μM. Samples at a given compound concentration were compared to the DMSO sample of the same DMSO concentration. Incubation with the compound was performed for 20 h (37 °C, 5% CO_2_). Subsequently, mitochondria were stained with Mito Tracker Deep Red (Thermo Fisher Scientific, Cat# M22426). The MitoTracker Deep Red stock solution (1 mM) was diluted to a final concentration of 100 nM in a prewarmed medium. The medium was removed from the plate leaving 10 μL residual volume and 25 μL of the Mito Tracker solution was added to each well. The plate was incubated for 30 min in darkness (37 °C, 5% CO_2_). To fix the cells 7 μL of 18.5% formaldehyde in PBS was added, resulting in a final formaldehyde concentration of 3.7%. Subsequently, the plate was incubated for another 20 min in darkness (RT) and washed three times with 70 μL of PBS. (Biotek Washer Elx405). Cells were permeabilized by the addition of 25 μL of 0.1% Triton X-100 to each well, followed by 15 min incubation (RT) in darkness. The cells were washed three times with PBS leaving a final volume of 10 μL. To each well, 25 μL of a staining solution was added, which contains 1% BSA, 5 μL/mL Phalloidin (Alexa594 conjugate, Thermo Fisher Scientific, A12381), 25 μg/mL Concanavalin A (Alexa488 conjugate, Thermo Fisher Scientific, Cat# C11252), and 5 μg/mL Hoechst-33342 (Sigma, Cat# B2261-25 mg), 1.5 μg/mL WGA-Alexa594 conjugate (Thermo Fisher Scientific, Cat# W11262) and 1.5 μM SYTO 14 solution (Thermo Fisher Scientific, Cat# S7576). The plate is incubated for 30 min (RT) in darkness and washed three times with 70 μL of PBS. After the final washing step, the PBS was not aspirated. The plates were sealed and centrifuged for 1 min at 500 rpm.

The plates were prepared in triplicates with shifted layouts to reduce plate effects and imaged using a Micro XL high-content screening system (Molecular Devices) in 5 channels (DAPI: Ex350–400/Em410–480; FITC: Ex470–500/Em510–540; Spectrum Gold: Ex520–545/Em560–585; TxRed: Ex535–585/Em600–650; Cy5: Ex605–650/Em670–715) with 9 sites per well and 20× magnification (binning 2).

The generated images were processed with the *CellProfiler* package (https://cellprofiler.org/, version 3.0.0)^70^ on a computing cluster of the Max Planck Society to extract 1716 cell features per microscope site. The data was then further aggregated as medians per well (9 sites → 1 well), then over the three replicates.

Further analysis was performed with custom *Python* (https://www.python.org/) scripts using the *Pandas* (https://pandas.pydata.org/) and *Dask* (https://dask.org/) data processing libraries as well as the *Scientific Python* (https://scipy.org/) package.

From the total set of 1716 features, a subset of highly reproducible and robust features was determined using the procedure described by Woehrmann et al.^71^ in the following way:

Two biological repeats of one plate containing reference compounds were analyzed. For every feature, its full profile over each whole plate was calculated. If the profiles from the two repeats showed a similarity ≥0.8 (see below), the feature was added to the set.

This procedure was only performed once and resulted in a set of 579 robust features out of the total of 1716 that was used for all further analyses.

The phenotypic profiles were compiled from the Z-scores of all individual cellular features, where the Z-score is a measure of how far away a data point is from a median value.

Specifically, Z-scores of test compounds were calculated relative to the median of DMSO controls. Thus, the Z-score of a test compound defines how many MADs (median absolute deviations) the measured value is away from the Median of the controls, as illustrated by the following formula:
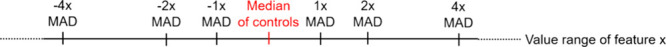

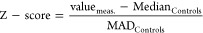


The phenotypic compound profile is then determined as the list of Z-scores of all features for one compound.

In addition to the phenotypic profile, an induction value was determined for each compound as the fraction of significantly changed features, in percent:
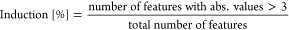


Similarities of phenotypic profiles (termed *Biosimilarity*) were calculated from the correlation distances (CD) between two profiles (https://docs.scipy.org/doc/scipy/reference/generated/scipy.spatial.distance.correlation.html):^72^
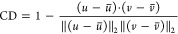
where *x̅* is the mean of the elements of *x*, *x* · *y* is the dot product of *x* and *y*, and ∥*x*∥_2_ is the Euclidean norm of *x*:
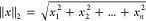


The Biosimilarity is then defined as
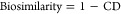


Biosimilarity values smaller than 0 are set to 0 and the Biosimilarity is expressed in percent (0–100).”

Compounds are considered active in CPA for induction ≥5% as above this value the profiles become stable and reproducible. Two profiles are considered biosimilar for biosimilarity ≥75% as for biosimilarity values <75%, the number of biosimilar profiles increases drastically because the comparisons become unspecific.

Compounds are usually screened first at 10 or 2 μM for reference compounds with low IC_50_ values for the annotated target. If compounds were inactive or had low induction values, higher concentrations were tested. If the induction values were too high, lower concentrations were tested.

### CPA Subprofile Analysis

Cluster subprofiles were generated as recently described.^14^ For a set of cluster-defining profiles, dominating features were extracted as follows: for each profile, the sign for each of the 579 features value was assessed. The counter for positive or negative values was determined. For all cluster-defining compounds, the maximum of the two counters was determined and divided by the total number of defining profiles. A given feature was added to the cluster profile if its value had the same sign (i.e., positive or negative feature values) for 85% of the defining profiles. Afterward, a representative median subprofile for the cluster was calculated by taking the median values over all cluster-defining profiles for every given feature and combining them into a new reduced profile. This median (*consensus*) subprofile was then used to calculate the biosimilarity of test compounds to the defined cluster subprofiles. Due to the shorter cluster subprofiles, the cluster biosimilarity threshold was set to 80%.

### MitoTracker Deep Red Staining

U-2OS cells were seeded at a density of 5000 cells/well into black 96-well plates with clear, flat bottom and incubated overnight at 37 °C and 5% CO_2_. The supernatant was exchanged for a test compound-containing medium, followed by 60 min of incubation at 37 °C and 5% CO_2_. MitoTracker Deep Red and Hoechst-33342 were added with final concentrations of 100 nM and 5 mg/mL, respectively. Cells were incubated for 3 min at 37 C and 5% CO_2_, rinsed twice with PBS, and fixed in 4% paraformaldehyde in PBS for 5 min at room temperature. Cells were imaged in PBS at 10× magnification using an Axiovert 200 M automated fluorescence microscope (Carl Zeiss, Germany). Stain intensities per cell were analyzed using MetaMorph 7.7.8.0. Data were normalized to the value for cells that were treated with DMSO, which was set to 100%.

### Visualization of the Mitochondrial Network

One day prior to compound treatment, 3 μL of CellLight Mitochondria-GFP BacMam 2.0 reagent was added per 1 × 10^4^ U-2OS cells directly to the complete medium. 3000 cells/well were seeded in 8-well ibidi chambers and incubated overnight at 37 °C and 5% CO_2_. The next day, the compounds were added to the cells and the fluorescence of live cells was recorded in real-time for 24 h using a confocal microscope SP5 Leica at excitation/emission 488/555 nm for 24 h at 37 °C and 5% CO_2_). The images and movies were analyzed using FIJI ImageJ version 1.52.^73^

### Real-Time Live-Cell Analysis

Cell growth and compound toxicity were observed by real-time live-cell analysis using an IncuCyte Zoom (Essen Bioscience); 5000 U-2OS cells were seeded per well in a black 96-well plate and incubated at 37 °C and 5% CO_2_ overnight. The medium was then exchanged with fresh medium containing the compounds or DMSO as a control. Propidium iodide and CellEvent Caspase-3/7 Green were also added to the medium to monitor the compound toxicity and apoptosis over time. Cells were incubated for 48 h and were imaged every hour. Cell confluence was quantified as a measure of cell growth using the IncuCyte ZOOM software (2018A). Red object confluence and green object confluence were quantified as a measure of PI-positive cells and caspase-3/7 activity, respectively.

### Proteome Profiling

One ×10^6^ U-2OS cells (1 × 10^6^) were seeded into a T75 flask. A day after, the medium was exchanged with DMEM-containing compound (10 and 30 μM ciclopirox; 2 μM GSK-J4, 30 μM SB-525334 and 30 μM compound **2**). DMSO was used as a control. After incubation at 37 °C and 5% CO_2_ for 24 h, the medium was removed and cells were washed with PBS. Cells were detached and washed twice with ice-cold PBS followed by centrifugation. Cells were resuspended in 1 mL PBS containing protease inhibitors and were lysed by freeze–thawing followed by centrifugation for 20 min at 15,000×*g*. Supernatants were collected, and protein concentration was determined using a DC assay; 75 μL of 2 g/L cell lysate was mixed with 75 of μL 200 mM triethylammonium bicarbonate (TEAB) buffer. After the addition of 7.5 μL of TCEP and incubation at 55 °C for 30 min, samples were treated with 7.5 μL of iodoacetamide (375 M) for 30 min in the dark. By adding 900 μL of prechilled acetone, proteins were precipitated and incubated at −20 °C overnight. The next day, samples were centrifuged for 10 min at 8000×*g* and 4 °C, and supernatants were removed. The dry protein pellet was dissolved in TEAB buffer containing trypsin, which was used according to the manufacturer’s protocol. After incubation overnight at 37 °C, samples were labeled with a TMT label according to the manufacturer’s instruction. All experiments were performed in biological triplicates.

Prior to nanoHPLC-MS/MS analysis, samples were fractionated into 10 fractions on a C18 column using high pH conditions to reduce the complexity of the samples and thereby increase the number of quantified proteins. Therefore, samples were dissolved in 120 μL of 20 mM ammonium formate (NH_4_COOH) at pH 11, followed by ultrasonication for 2 min, subsequent vortexing for 1 min, and centrifugation at 8000×*g* for 3 min at room temperature. 50 μL of the C18 supernatant was injected onto an XBridge C18 column (130 Å, 3.5 μm, 1 mm × 150 mm) using a U3000 capHPLC system (ThermoFisher Scientific, Germany). Separation was performed at a flow rate of 50 μL/min using 20 mM NH_4_COO pH 11 in water as solvent A and 40% 20 mM NH_4_COO pH 11 in water premixed with 60% acetonitrile as solvent B. Separation conditions were 95% solvent A/5% solvent B isocratic for the first 10 min, to desalt the samples, followed by a linear gradient up to 25% in 5 min, a second linear gradient up to 65% solvent B in 60 min, and a third linear gradient up to 100% B in 10 min. Afterward, the column was washed at 100% solvent B for 14 min and re-equilibrated to starting conditions. Detection was carried out at a valve length of 214 nm. The eluate between 15 and 100 min was fractionated into 10 fractions (30 s per fraction, circular fractionation using 10 vials). Each fraction was dried in a SpeedVac at 30 °C until complete dryness and subsequently subjected to nanoHPLC-MS/MS analysis.

For nanoHPLC-MS/MS analysis, samples were dissolved in 20 μL of 0.1% TFA in water and 1–3 μL were injected onto an UltiMateTM 3000 RSLCnano system (ThermoFisher Scientific, Germany) online coupled to a Q Exactive HF Hybrid Quadrupole-Orbitrap Mass Spectrometer equipped with a nanospray source (Nanospray Flex Ion Source, Thermo Scientific). All solvents were of LC-MS grade. To desalt the samples, they were injected onto a precolumn cartridge (5 μm, 100 Å, 300 μm ID × 5 mm, Dionex, Germany) using 0.1% TFA in water as eluent with a flow rate of 30 μL/min. Desalting was performed for 5 min with eluent flow to waste followed by back-flushing of the sample during the whole analysis from the precolumn to the PepMap100 RSLC C18 nano-HPLC column (2 μm, 100 Å, 75 μm ID × 50 cm, nanoViper, Dionex, Germany) using a linear gradient starting with 95% solvent A (water containing 0.1% formic acid)/5% solvent B (acetonitrile containing 0.1% formic acid) and increasing to 60% solvent A 0.1% formic acid/40% solvent B in 120 min using a flow rate of 300 nL/min. Afterward, the column was washed (95% solvent B as the highest acetonitrile concentration) and re-equilibrated to starting conditions. The nano-HPLC was online coupled to the quadrupole-orbitrap mass spectrometer using a standard coated SilicaTip (ID 20 μm, Tip-ID 10 μM, New Objective, Woburn, MA, USA).

A mass range of *m*/*z* 300 to 1650 was acquired with a resolution of 60,000 for a full scan, followed by up to 15 high energy collision dissociation (HCD) MS/MS scans of the most intense at least doubly charged ions using a resolution of 30,000 and an NCE energy of 35%. Data evaluation was performed using MaxQuant software (1.6.17.0).^74^ including the Andromeda search algorithm and searching the human reference proteome of the Uniprot database. The search was performed for full enzymatic trypsin cleavages, allowing two miscleavages. For protein modifications, carbamidomethylation was chosen as fixed, and oxidation of methionine and acetylation of the N-terminus were chosen as variable modifications. For relative quantification, the type “reporter ion MS2” was chosen, and for all lysines and peptide N-termini, TMT labels were defined. The mass accuracy for full mass spectra was set to 20 ppm (first search) and 4.5 ppm (second search), respectively, and for MS/MS spectra to 20 ppm. The false discovery rates for peptide and protein identification were set to 1%. Only proteins for which at least two peptides were quantified were chosen for further validation. Relative quantification of proteins was carried out using the reporter ion MS2 algorithm implemented in MaxQuant. The proteinGroups.txt file was used for further analysis. All proteins that were not identified with at least two razor and unique peptides in at least one biological replicate were filtered off. The replicates were grouped together, and all proteins not quantified in at least three replicates in at least one of the groups (treated or control, respectively) were filtered off. Afterward, these values were normalized to the median, and a two-sides *t*-test was performed. Only proteins with a *p* value < 0.03 and *p* value > −0.03 were considered as statistically significantly up- or down-regulated. For compound SB525334 both *p* value < 0.03 and *p* value > −0.03 and *p* value < 0.02 and *p* value > −0.02 were considered. Pathway-over representation analysis was performed using the QIAGEN Ingenuity Pathway Analysis tool (IPA).^75^ Volcano plots were generated using the web-based tool VolcaNoseR.^76^

### Seahorse Cell Mito Stress Test

The Cell Mito Stress Test kit was performed using a Seahorse XFp analyzer (Agilent, USA) according to the manufacturer’s protocol. Two ×10^5^ U-2OS cells were seeded per wells into an XFp cell culture mini plate and incubated overnight at 37 °C, 5% CO_2_. Using the XF Calibrant, the XFp cartridges were hydrated and incubated overnight at 37 °C. The next day, the cell medium was exchanged to pH 7.4 DMEM-based assay medium (Agilent, USA) containing 2 mM GlutaMAX (ThermoFisher), 1 mM sodium pyruvate (PAN Biotech, Germany), and 25 mM glucose (Sigma-Aldrich, Germany). After five measurements of baseline recording, the test compounds were injected, followed by ten measurement intervals. Subsequently, oligomycin A, FCCP, and rotenone/antimycin A were injected, followed by three measurement intervals after each injection. For compound pretreatment, the test compound was added 24 h before the assay to the XFp cell culture plates. The background was subtracted from all data and values were normalized to the last baseline measurement (which was set to 100%) using the Wave software Version 2.6.0 (Agilent, USA). The results were plotted using GraphPad Prism 9 software.

### Analysis of the Mitochondrial Membrane Potential

5 × 10^3^ U-2OS cells per well were seeded in a black 96-well plate with a clear flat bottom and incubated for 24 h at 37 °C and 5% CO_2_. Afterward, the seeding medium was replaced with a medium containing the compounds. After incubation for 24 h at 37 °C and 5% CO_2_, 20 μM FCCP or 0.5% DMSO was added as controls. Cells were incubated at 37 °C and 5% CO_2_ for 10 more minutes. The staining solution was prepared by adding 1 μM TMRE and 5 μg/mL Hoechst-33342 to DMEM supplemented with 4 mM l-glutamine, 10% fetal bovine serum, 1 mM sodium pyruvate, and nonessential amino acids. After removing the medium from the cells, the staining solution was added, and the cells were incubated for 30 min at 37 °C and 5% CO_2_. Then, the solution was removed, and the cells were washed twice with PBS before 0.2% BSA in PBS was added. TMRE fluorescence was recorded using the Axiovert 200 M automated microscope (Carl Zeiss, Germany) with an excitation/emission wavelength of 549 nm/575 nm for TMRE and 361 nm/497 nm for Hoechst-33342 at a 10-fold magnification and at 37 °C and 5% CO_2_. The obtained images were analyzed using the Multi Wavelength Cell Scoring function of the Meta Morph software version 7.7.8.0 and the results were plotted using GraphPad Prism 9 software.

### MitoSOX Red Assay

Mitochondrial superoxide levels were determined using the indicator dye MitoSOX Red (Thermo Fisher, USA); 15,000 U-2OS were seeded per well into black 96-well plates with clear flat bottom and incubated at 37 °C and 5% CO_2_ overnight. The seeding medium was exchanged for a staining medium comprising DMEM without additives and containing 5 μM MitoSOX Red and 5 μg/μL Hoechst-33342 (ThermoFisher, USA). Cells were incubated for 30 min at 37 °C and 5% CO_2_. Subsequently, the medium was exchanged for DMEM with additives containing the test compounds, followed by 60 min of incubation at 37 °C and 5% CO_2_. Cells were fixed in PBS containing 0.5% paraformaldehyde for 10 min at room temperature and washed three times with PBS. Cells were imaged using an Axiovert 200 M automated microscope (Carl Zeiss, Germany) at 10× magnification. MetaMorph 7.7.8.0 (Visitron, Germany) was used to quantify the integrated fluorescence intensity of MitoSOX Red per cells. The data were normalized to control cells treated with either DMSO (set to 0%) or 10 μM 1-chloro-2,4-dinitrobenzene (CDNB) (set to 100%). The results were plotted using GraphPad Prism 9 software.

### Reverse Transcriptase-Quantitative PCR (RT-qPCR)

U-2OS cells were seeded into six-well plates (1 × 10^5^ cells/well) and incubated for 24 h until they reached approximately 80% confluence. Cells were then treated with the compounds or DMSO as a control for 24 h. The total RNA was isolated using the RNAeasy Kit (Qiagen, no. 74104) including the DNase digestion step. The concentration of RNA was determined by using the RNA BR assay kit from (ThermoFisher, no. Q10210) in conjunction with the Qubit4.0 (ThermoFisher). cDNA was obtained using the QuantiTect Reverse Transcription Kit (Qiagen, #205313). The relative mRNA amount of the *ATF4* gene was evaluated using the SsoAdvanced Universal SYBR Green Supermix (Bio-Rad, #1725274) using the CFX96 Real-Time PCR Detection System (Bio-Rad, Germany). Relative expression levels were calculated using the ΔΔCt method,^77^ using *GAPDH* as a reference gene. Gene expression levels for samples that were treated with DMSO were set to 100%. The results were plotted using GraphPad Prism 9 software. Employed primer pairs (obtained from Sigma-Aldrich) were as follows: Human-ATF4 (NM_001675) fw: 5′- TTCTCCAGCGACAAGGCTAAGG-3′, rv: 5′- CTCCAACATCCAATCTGTCCCG-3′. Human-GAPDH (NM_002046) fw: 5′- GTCTCCTCTGACTTCAACAGCG-3′, rv: 5′- ACCACCCTGTTGCTGTAGCCAA-3′.

### Iron Chelation Assay

Compounds were incubated with 12.5 μM Fe(II) (FeSO_4_) at room temperature for 10 min in a clear 96-well white plate. DMSO, deferoxamine, and EDTA were used as controls. Afterward, 0.5 mM ferrozine was added to the solution, and the absorbance at 561 nm was detected using a TecanSpark Microplate Reader. The results were plotted using GraphPad Prism 9 software.

### HIF1-α Reporter Gene Assay

HEK-293T cells were transfected with the pGL4.22-PGK1-HRE::dLUC plasmid (the plasmid was a gift from Chi Van Dang (Addgene plasmid# 128095; http://n2t.net/addgene:128095; RRID:Addgene_128095))^100^ and pRL-TK plasmid for constitutive expression of *Renilla* luciferase (Promega, Cat# E2241). The plasmids were diluted in Opti-MEM and 3 μL per μg plasmid Lipofectamine 2000 transfection reagent was added to the solution and incubated for 15 min. The transfection mixture was added to 2.78 × 10^5^ cells/mL and 2.5 × 10^4^ cells/well were seeded into white 96-well plates (Corning, #353075). After incubation overnight, cells were treated with the compounds or 100 μM CoCl_2_ and 10 μM ML228 as controls for 24 h. Luciferase activities were determined using the Dual-Glo Luciferase Assay System (Promega, Cat# E2940). Values obtained for firefly luciferase were normalized to the corresponding *Renilla* luciferase values. Results are shown as fold induction determined upon normalization to the DMSO control. The results were plotted using GraphPad Prism 9 software.

### Immunoblotting

For quantification of the HIF1-α and ATF4 protein levels, U-2OS cells were seeded into 6-well plates and incubated until they reached a confluence of 80%. Cells were treated with different concentrations of the compounds or DMSO as a control. After incubation for 24 h at 37 °C and 5% CO_2_, cells were washed with PBS followed by detachment using a cell dissociation solution (Gibco, Cat#13151-014) for 10 min at 37 °C. Detached cells were collected in 1.5 mL low-binding Eppendorf tubes (Eppendorf, #0030108116). Samples were then centrifuged for 3 min at 340×*g*, and the cell pellets were washed with ice-cold PBS. For HIF1-α, cells were suspended in a lysis buffer (0.01% bromphenol blue, 10% glycerol, 20% SDS, 62.5 mM Tris (pH 6.8), and 5% 2-mercaptoethanol). For the detection of ATF4, cells were lysed in RIPA buffer. Three freeze–thaw cycles were performed to lyse the cells. Samples were centrifuged at 16,000×*g* and 4 °C for 30 min, supernatants were transferred to fresh low-binding Eppendorf tubes, and protein concentrations were determined using the DC protein assay according to the instructions of the manufacturer. Proteins were separated via SDS-PAGE and using wet blotting proteins were transferred onto a polyvinylidene difluoride (PVDF) membrane. Membranes were stained for HIF1-α (BD Biosciences, Cat# 610959, 1:500 dilution), and β-actin as a control (Abcam, Cat# ab8227). For quantification of ATF4 levels, membranes were stained with an ATF4 antibody (Cell Signaling, Cat# 11815, 1:1000) and an antivinculin antibody (Sigma-Aldrich, Cat# V9131) as a control. Secondary antibodies coupled to IR dye800CW and IR dye680RD were employed for the detection of HIF1-α, β-actin, and vinculin, whereas ATF4 was detected using horseradish peroxidase (HRP)-coupled antibody. Membranes were imaged using the ChemiDoc MP Imaging System (BioRad). Quantification of band intensities was performed using Image Lab Version 5.2 (BioRad).

### Purity of the Compounds

Two different methods were employed to examine the purities of the compounds. Purities were determined on either an UHPLC-System (1290 Infinity II LC System; Agilent) utilizing a filter column (Ghost-Guard-LC 30 × 4.6 mm; MZ-Analysentechnik) or a column (Poroshell 120 EC-C18, 1.9 μm, 2.1 × 50 mm; Agilent) with upstream precolumn (Poroshell 120 EC-C18, 3 × 5 mm, 2.7 μm; Agilent). All samples were analyzed using a qualitative method (Software: MassHunter Workstation V.10; Agilent). Peaks that were not captured by the integration algorithm were integrated manually; the analyzed samples were generated as single sample reports in the PDF format (Software: MassHunter Analytical Studio Reviewer V. B.02.01, Agilent). Purity values are determined from the area percent of the total wave chromatogram (TWC). Alternatively, purities were determined using an HPLC-System (Performance System; Shimadzu) utilizing a column (Shim-Pack XR-ODS, 2.2 μm, 2.0 mm × 50 mm, Shimadzu). Solvents used in both methods were LC-grade solvents and ultrapure H_2_O. Mobile phases were A (H_2_O + 0.1% FA (v/v)) and B (ACN + 0.1%FA (v/v)). All samples were analyzed using a qualitative Method (Software: LabSolution V5, Shimadzu), and the analyzed samples were generated as single sample reports in PDF format (Software: OpenSolution V1, Shimadzu). Purity values are determined from the area percent of UV 210 nm.

The purity of the explored compounds was >95%. Dephostatin, rotenone, and ML228 had purities of 88, 84, and 91%, respectively.

### Pan-Assay Interference

All compounds used in this study besides pyrvinium pamoate, FCCP, and PAC-1 do not contain PAINS^78^ (determined using PAINS filters;^79,80^ PAINS filters were implemented with Pipeline Pilot (BioVia).

### Quantification and Statistical Analysis

All biological replicates were either representative of three independent (biological) replicates or expressed as mean ± SD. All statistical details of the conducted experiments can be found in the respective figure and table legends. n: number of biological replicates.

## Data Availability

Further information and requests for resources and reagents should be directed to and will be fulfilled by the corresponding author, Slava Ziegler (slava.ziegler@mpi-dortmund.mpg.de). The proteome data sets generated during this study are available at MassIVE (MSV000093287). The biosimilarity to the MitoStress cluster for reference compounds can be accessed via the web app tool https://cpcse.pythonanywhere.com/.

## Supporting Information Available

U-2OS cells treated with 0.5% DMSO as a control (AVI)
U-2OS cells treated with 30 μM ciclopirox for 24 h (AVI)
U-2OS cells treated with 2 μM GSK-J4 for 24 h (AVI)
CPA profiles for DFO and phenanthroline (Figure S1), profile comparison for ciclopirox related to the individual stains (Figure S2), profile analysis for ETC inhibitors (Figure S3), CPA profiles for SB525334 and compound **2** and dose-dependent change of MitoTracker-related features (Figure S4), influence of the compounds on cell growth (Figure S5) or the mitochondrial membrane potential (Figure S6), Volcano plots from the proteome profiling for GSK-J4 and SB525334 (Figure S7), influence in HIF signaling and iron chelation (Figure S8), uncropped blots (Figure S9), cluster subprofiles for the 13 defined clusters (Figure S10), cluster biosimilarities for DFO, phenanthroline, deferasirox, PAC-1 and Sal003 (Figure S11), cluster biosimilarities for compounds studies for mitotoxicity (Figure S12), profile analysis for salubrinal (Figure S13), profile analysis for ponatinib (Figure S14), compounds biosimilar to ciclopirox (Table S2), compounds biosimilar to ciclopirox when only mitochondrial features are considered (Table S3), overlap of downregulated proteins in Quiros et al. and this study (Table S7), upregulated genes and proteins in Quiros et al. and this study (Table S8), MitoStress defining compounds (Table S9) (PDF)
CPA features for cicloprox (Table S1); proteins regulated by CPX 10 and 30 μM (Table S4); proteome profiling data (Table S5); and ingenuity pathways analysis analysis (Table S6) (XLSX)
Molecular formula strings (CSV)

## Supplementary Material

jm4c01183_si_001.avi

jm4c01183_si_002.avi

jm4c01183_si_003.avi

## ABBREVIATIONS

ALK5: activin receptor-like kinase 5
AMA: antimycin A
ATF4: cyclic AMP-dependent transcription factor 4
Biosim: biosimilarity
CDNB: 1-chloro-2,4-dinitrobezene
ConA: concanavalin A
Conc: concentration
CPA: Cell Painting assay
Cpd: compound
CPX: ciclopirox
DFO: deferoxamine
DHODH: dihydroorotate dehydrogenase
DLBL: diffuse large B-cell lymphoma
DMEM: Dulbecco’s modified Eagle’s medium
ECAR: extracellular acidification rate
EGLN1: HIF1 prolyl hydroxylase Egl nine homolog
EIF2: eukaryotic translation initiation factor 2
Em: emission
ETC: electron transport chain
Ex: excitation
FCCP: carbonylcyanid-4-(trifluormethoxy)phenylhydrazon
Gal: galactose
GAPDH: glyceraldehyde-3-phosphate dehydrogenase
Glu: glucose
GLUT: glucose transporter
HIF: hypoxia inducible factor
HK2: hexokinase-2
HMOX1: heme oxygenase 1
HRP: horseradish peroxidase
Ind: induction
ISR: integrated stress response
JMJD3: JmjC domain-containing protein 3
KDM6B: lysine-specific demethylase 6B
L/CH: lysosmotropism/cholesterol homeostasis
MAD: median absolute deviation
MEM: minimal essential medium
MoA: mode of action
MRP: mitochondrial ribosomal protein
NDUF: NADH-ubiquinone oxidoreductase subunit
OCR: oxygen consumption rate
OMA: oligomycin A
OXPHOS: oxidative phosphorylation
PAIN: pan-assay interference
PHD: prolyl hydroxylase domain
Phe: phenanthroline
PI: propidium iodide
PVDF: polyvinylidene difluoride
PYR: pyrimidine
ROT: rotenone
RIPA: radio-immunoprecipitation assay
RT-qPCR: reverse transcriptase-quantitative PCR
SD: standard deviation
SLC2A1: solute carrier family 2 member
TCA: tricarboxylic acid cycle
TCEP: tris(2-carboxyethyl)phosphine
TMRE: tetramethylrhodamine, ethyl ester
TEAB: triethylammonium bicarbonate
TMT: tandem mass tag
UMAP: uniform manifold approximation and projection
uORF: upstream open reading frame
VHL: Von Hippel-Lindau disease tumor suppressor
WGA: wheat germ agglutinin
