# Supplementary material for: Detection of a Mitochondrial Fragmentation and Integrated Stress Response Using the Cell Painting Assay
Source: J Med Chem. 2024 Jul 17;67(15):13252–70. doi: 10.1021/acs.jmedchem.4c01183 (PMC11320566; doi:10.1021/acs.jmedchem.4c01183)
Supplement: Supplementary file 4 — jm4c01183_si_004.pdf [file jm4c01183_si_004.pdf]

Supporting Information

Detection of a Mitochondrial Fragmentation and Integrated Stress Response Using the Cell Painting Assay

Soheila Rezaei Adariani[1,3], Daya Agne[1], Sandra Koska[1], Annina Burhop[1], Carina Seitz[2], Jens Warmers[1,3], Petra Janning[1], Malte Metz[1], Axel Pahl[2], Sonja Sievers[2], Herbert Waldmann[1,3] and Slava Ziegler[1]*

1 *Max Planck Institute of Molecular Physiology, Department of Chemical Biology, Otto-Hahn-Strasse 11, Dortmund 44227 (Germany)*

2 *Max Planck Institute of Molecular Physiology, Compound Management and Screening Center, Otto-Hahn-Strasse 11, Dortmund 44227 (Germany)*

3 *Technical University Dortmund, Faculty of Chemistry and Chemical Biology, Otto-Hahn-Strasse 6, Dortmund 44227 (Germany)*

*Correspondence: slava.ziegler@mpi-dortmund.mpg.de

Supporting Information

Table of Contents

Supporting Figures....S3

Supporting Tables....S16

Supporting Movies....S25

HPLC traces....S26

References....S30

Supporting Figures

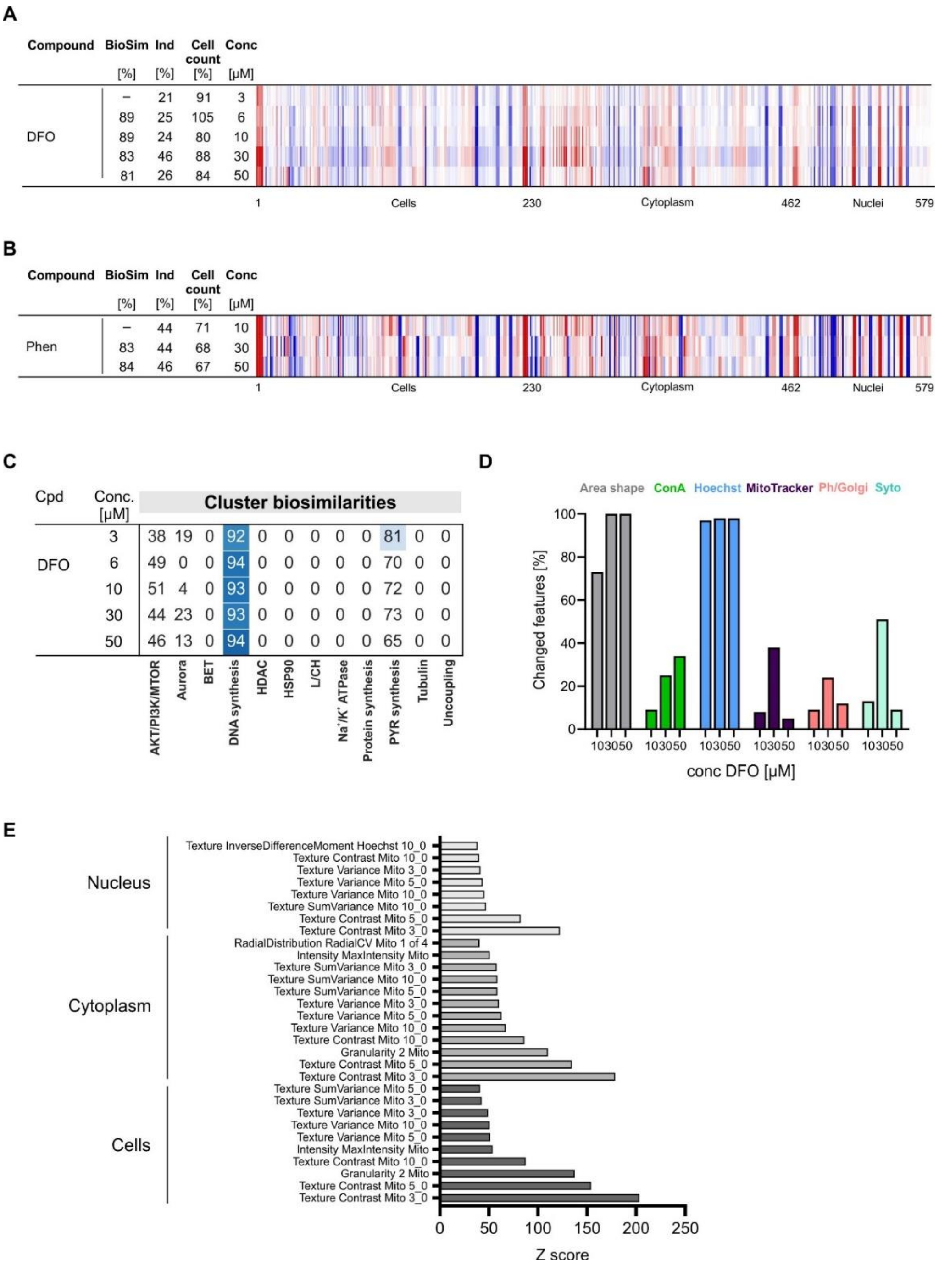

Figure S1 (related to Figure 1): CPA profiles for deferoxamine (DFO) and phenanthroline. (A and B) Comparison of the profiles for DFO (A) and phenanthroline (B) at different concentrations. The top line profile is set as a reference profile (100 % biological similarity, BioSim) to which the following profiles are compared. Blue color: decreased feature; red color: increased feature. (C) Cluster biosimilarity heatmap for DFO. Percent values are displayed. (D) Dose-dependent change in dye-related CPA features at different concentrations DFO. (E) Z scores for top 30 altered features with high Z scores for ciclopirox at 30 µM as determined in CPA. Cpd: compound; BioSim: biosimilarity; Ind: induction; Conc: concentration.

Compound	BioSim [%]	Ind [%]	Cell count [%]	Conc [µM]
CPX	–	59	75	30
ML228	94	66	71	3
ML228	91	54	65	10
SC144	92	52	69	2
GSK-J4	81	46	80	10
GSK-J4	79	41	85	2
JIB-04	78	45	79	2
Sanguinarine	70	36	71	1
Chelerythrine	71	33	86	30
cpd **1**	68	30	92	2
Pyrvinium	74	44	69	1

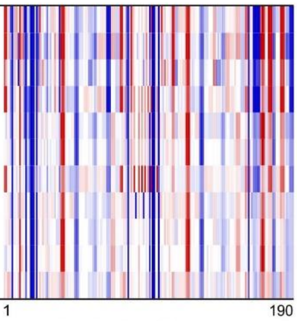

Compound	BioSim [%]	Ind [%]	Cell count [%]	Conc [µM]
CPX	–	59	75	30
ML228	97	66	71	3
ML228	95	54	65	10
SC144	94	52	69	2
GSK-J4	78	46	80	10
GSK-J4	78	41	85	2
JIB-04	76	45	79	2
Sanguinarine	59	36	71	1
Chelerythrine	54	33	86	30
cpd **1**	49	30	92	2
Pyrvinium	68	44	69	1

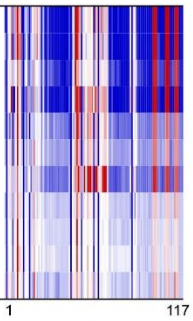

Compound	BioSim [%]	Ind [%]	Cell count [%]	Conc [µM]
CPX	–	59	75	30
ML228	90	66	71	3
ML228	91	54	65	10
SC144	85	52	69	2
GSK-J4	93	46	80	10
GSK-J4	87	41	85	2
JIB-04	63	45	79	2
Sanguinarine	83	36	71	1
Chelerythrine	86	33	86	30
cpd **1**	90	30	92	2
Pyrvinium	86	44	69	1

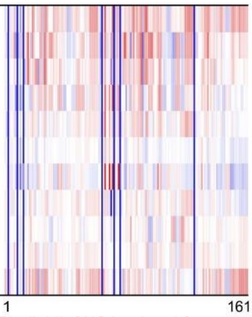

Compound	BioSim [%]	Ind [%]	Cell count [%]	Conc [µM]
CPX	–	59	75	30
ML228	92	66	71	3
ML228	83	54	65	10
SC144	85	52	69	2
GSK-J4	84	46	80	10
GSK-J4	90	41	85	2
JIB-04	70	45	79	2
Sanguinarine	71	36	71	1
Chelerythrine	91	33	86	30
cpd **1**	92	30	92	2
Pyrvinium	77	44	69	1

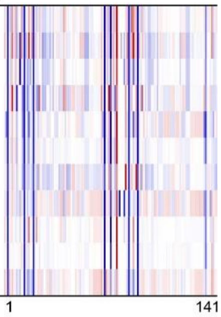

Figure S2 (related to Figure 2): Profile comparison for ciclopirox (CPX) and biosimilar compounds considering the features related to each stain only.

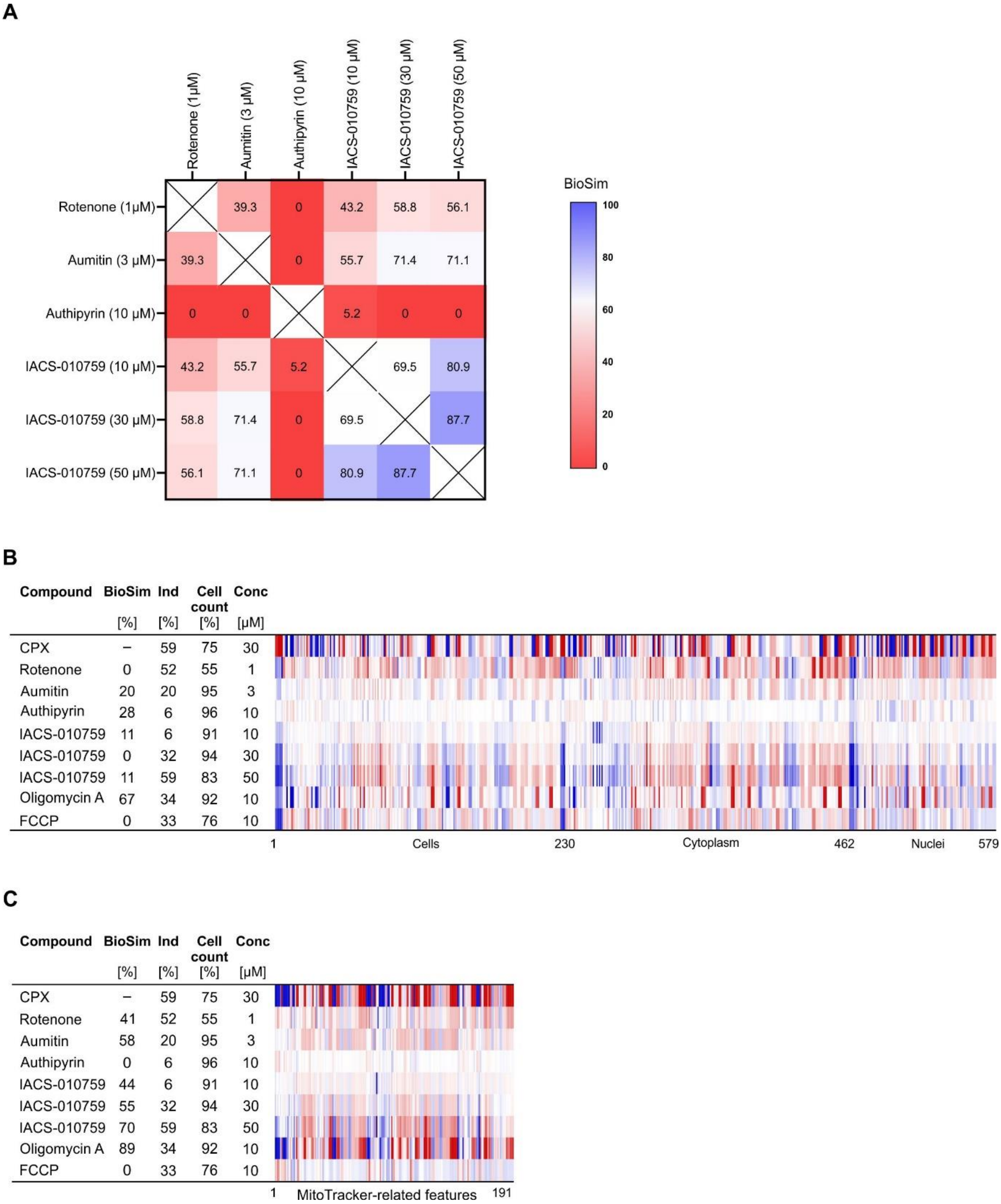

Figure S3 (related to Figure 2): Profile analysis for inhibitors of the mitochondrial electron transport chain (ETC). (A) Profile cross-similarity for ETC inhibitors. (B) Comparison of the profile of ciclopirox (CPX) at 30 µM to the profiles of ETC inhibitors. (C) Comparison of the profiles for ciclopirox (CPX) at 30 µM to the profiles of ETC inhibitors considering only MitoTracker-related features. For B and C: the top line profile is set as a reference profile (100 % biological similarity, BioSim) to which the following profiles are compared. Blue color: decreased feature; red color: increased feature. Cpd: compound; BioSim: biosimilarity; Ind: induction; Conc: concentration.

A

Cpd	Conc [µM]	AKT/PI3K/MTOR	Aurora	BET	DNA synthesis	HDAC	HSP90	L/CH	Na^+/K^+ ATPase	Protein synthesis	PYR synthesis	Tubulin	Uncoupling
		**Cluster biosimilarities**											
	0.2	0	0	51	0	54	0	15	65	28	0	46	16
	2	0	5	73	0	74	0	46	64	52	0	68	28
SB525334	10	0	0	31	0	31	24	42	28	26	0	39	9
	30	0	0	33	0	32	27	44	23	29	0	41	2
	50	0	0	30	0	31	33	41	19	32	0	39	2

B

Cpd	Conc [µM]	AKT/PI3K/MTOR	Aurora	BET	DNA synthesis	HDAC	HSP90	L/CH	Na^+/K^+ ATPase	Protein synthesis	PYR synthesis	Tubulin	Uncoupling
		**Cluster biosimilarities**											
cpd **2**	10	11	0	0	34	0	31	21	0	8	13	16	0

C

Compound	BioSim [%]	Ind [%]	Cell count [%]	Conc [µM]
CPX	–	59	75	30
cpd **2**	79	34	95	10

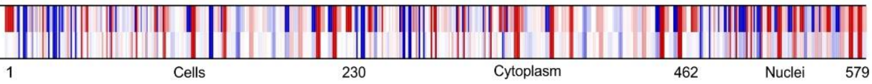

D

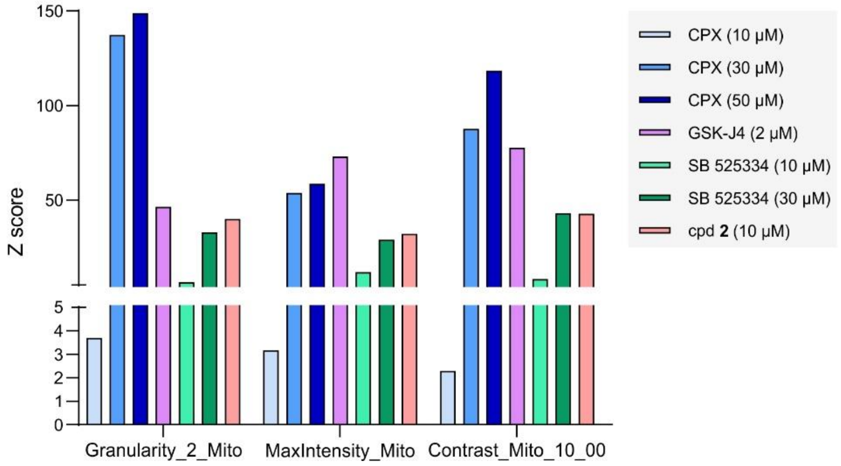

Figure S4 (related to Figure 2): CPA profile for SB525334 and compound 2. (A and B) Cluster biosimilarity heatmap for SB525334 (A) and compound **2** (B) Values are biosimilarity in %. (C) Comparison of the profile of ciclopirox (CPX) at 30 µM to the profile of compound **2** at 10 µM. The top line profile is set as a reference profile (100 % biological similarity, BioSim) to which the following profile is compared. Blue color: decreased feature; red color: increased feature. Cpd: compound; BioSim: biosimilarity; Ind: induction; Conc: concentration. L/CH: Lysosomotropism/cholesterol homeostasis; PYR: pyrimidine. (D) Quantification of the MitoTracker staining in CPA. Z scores for selected cells-related features are shown.

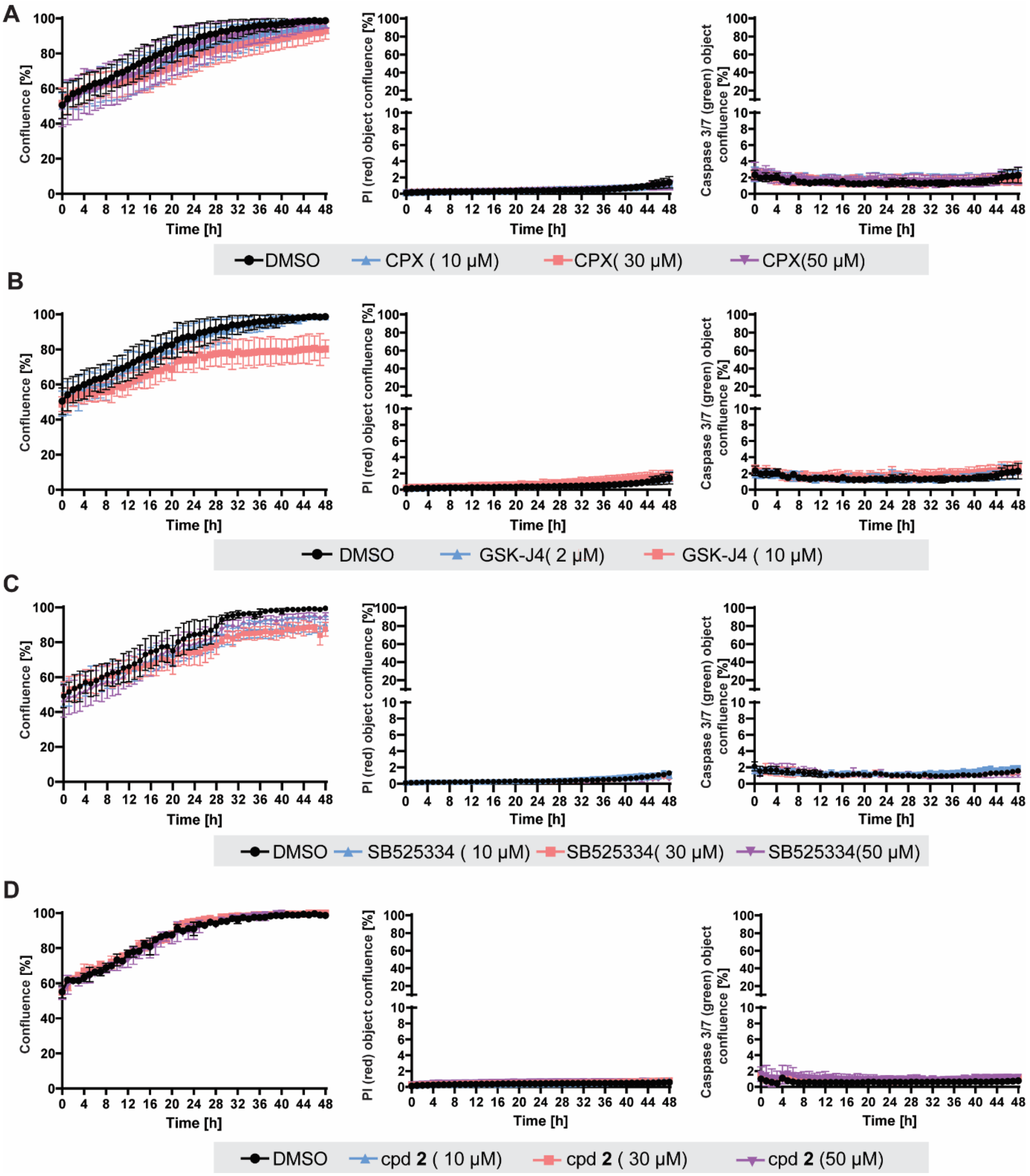

Figure S5: Influence of the compounds on cell growth. U-2OS cells were treated with the compounds for 48 h in presence of propidium iodide (PI) and CellEvent™ Caspase-3/7 Green to detect cell toxicity and apoptosis, respectively. Images were acquired every hour using the IncuCyte Zoom imaging system. Image-based analysis was performed to quantify cell growth by means of cell confluence as a readout, or cell toxicity and apoptosis by means of PI and caspase 3/7 activity-related fluorescence. (n =3; mean values ± SD). (A) Ciclopirox. (B) GSK-J4. (C) SB525334. (D) Compound **2**.

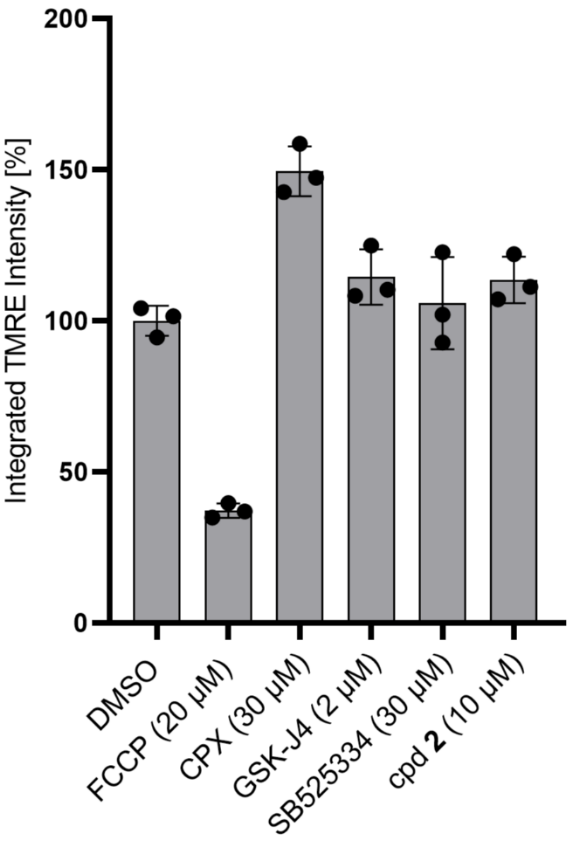

Figure S6: Influence on the mitochondrial membrane potential. U-2OS cells were treated with the compounds for 24 h prior to the addition of tetramethylrhodamine, methyl ester (TMRE) to determine mitochondrial membrane potential. FCCP was used as a positive control (n =3; mean values ± SD).

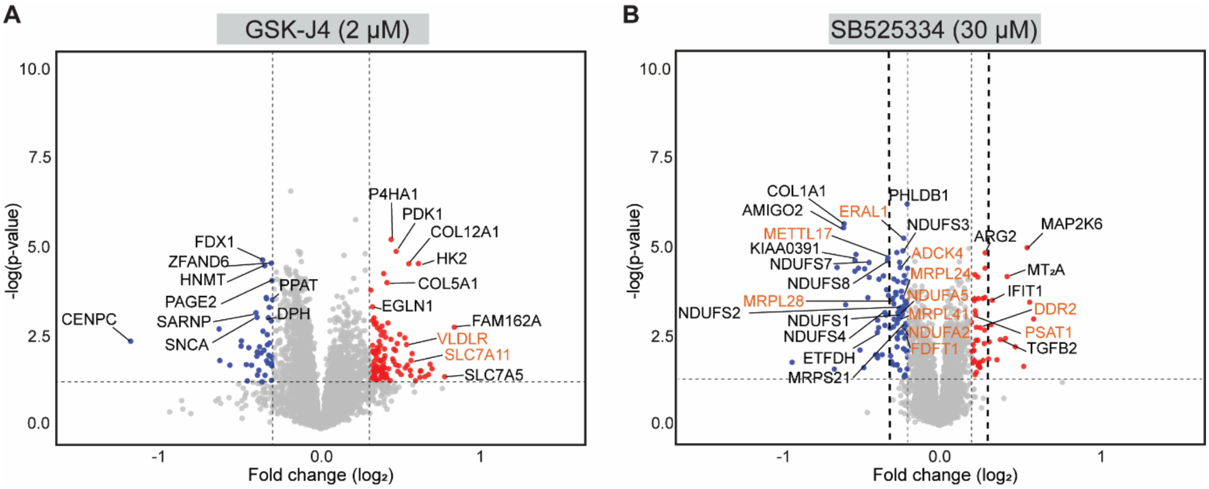

Figure S7 (related to figure 5): Proteomics analysis. Volcano plot of log2 fold changes in protein abundance upon treatment for 24 h with 2 µM GSK-J4 (A) or 30 µM SB525334 (B) (FC <±0.2; light gray; FC <±0.3; black). Red circles: upregulated proteins; blue circles: downregulated proteins. orange circles: proteins found regulated in Quiros et al. Volcano plots were visualized using VolcaNoseR[1]. FC: fold change.

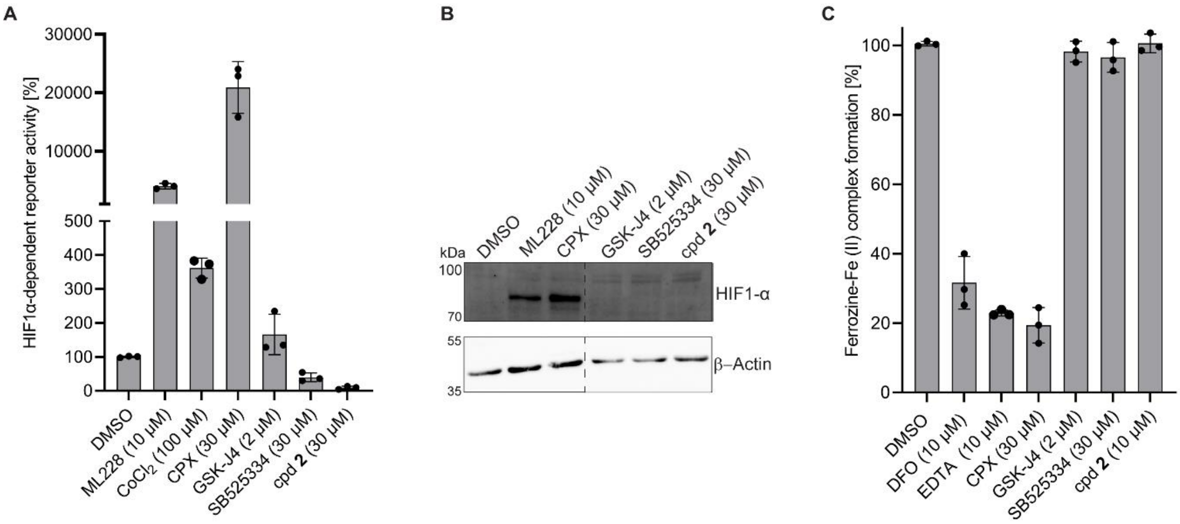

Figure S8 (related to Figure 5): Influence of the compounds on HIF1 signaling and iron chelation. (A) HIF1-α-dependent reporter gene assay. HEK293 cells transfected with pGL4.22-PGK1-HRE::dLUC and *Renilla* luciferase-expressing plasmids were treated with the compounds or DMSO as a control for 24 h prior to detection of firefly and *Renilla* luciferase activities. ML228 and $CoCl_2$ were used as controls for HIF1 induction. Mean values ± SD (n =3). (B) Detection of HIF1-α protein levels in U-2OS cells after treatment with the compounds for 24 h. Cells were treated with the compounds prior to detection of HIF1-α and β-actin as a reference protein using immunoblotting. Representative blot is shown (n =3). Lanes in the immunoblot were rearranged to fit the figure. See Figure S9 for the full blots. (C) Using Ferrozine-Fe(II) complex formation to determine iron chelation by the compounds. Mean values ± SD (n =3).

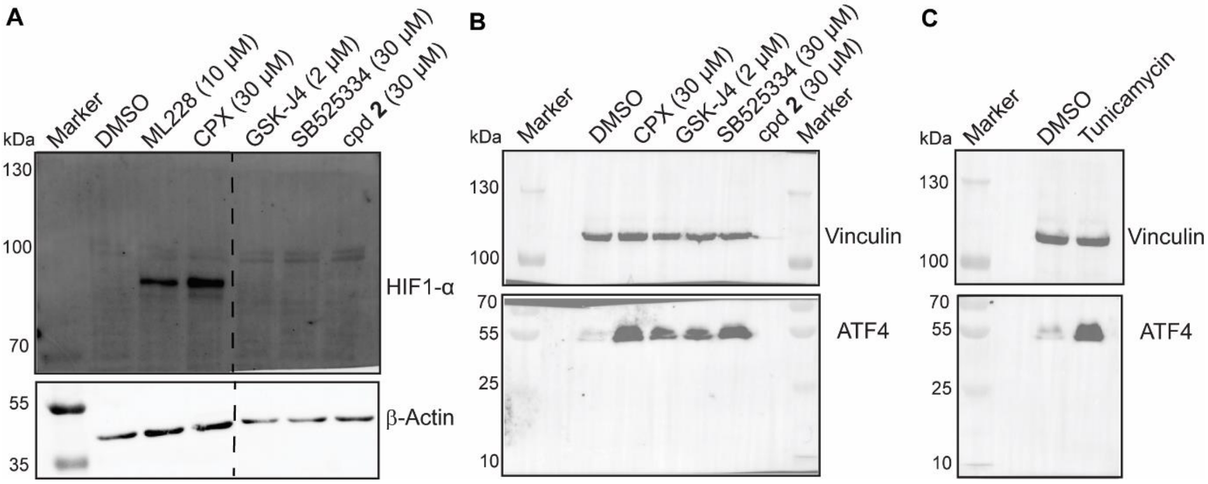

Figure S9: Full blots for the results shown in Figure. Figure S8B (A), 6D (B) and 6E (C).

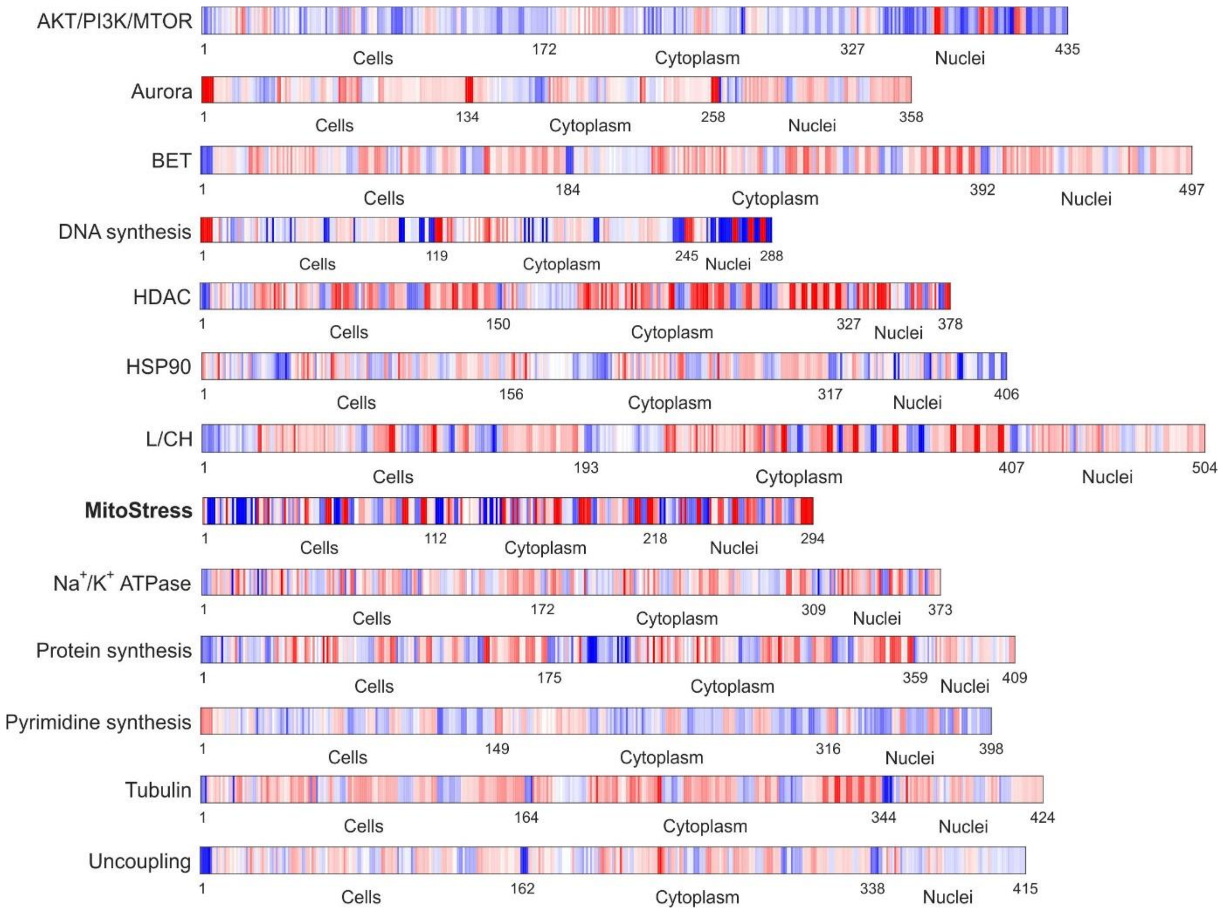

Figure S10 (related to Figure 7): Median cluster subprofiles of the 13 defined clusters. The median cluster subprofiles for all clusters besides MitoStress were previously reported [2]. Blue color: decreased feature, red color: increased feature.

A

Cpd	Conc [µM]	AKT/PI3K/MTOR	Aurora	BET	DNA synthesis	HDAC	HSP90	L/CH	MitoStress	Na+/K+ ATPase	Protein synthesis	PYR synthesis	Tubulin	Uncoupling
DFO	3	38	19	0	92	0	0	0	43	0	0	81	0	0
DFO	6	49	0	0	94	0	0	0	39	0	0	71	0	0
DFO	10	51	4	0	93	0	0	0	35	0	0	72	0	0
DFO	30	44	23	0	93	0	0	0	20	0	0	73	0	0
DFO	50	46	13	0	94	0	0	0	30	0	0	65	0	0

Cluster biosimilarities

B

Cpd	Conc [µM]	AKT/PI3K/MTOR	Aurora	BET	DNA synthesis	HDAC	HSP90	L/CH	MitoStress	Na+/K+ ATPase	Protein synthesis	PYR synthesis	Tubulin	Uncoupling
Phen	10	46	12	0	96	0	0	0	31	0	0	61	0	0
Phen	30	44	17	0	96	0	0	0	63	0	0	60	0	0
Phen	50	48	8	0	93	0	0	0	62	0	0	61	0	0

Cluster biosimilarities

C

Cpd	Conc [µM]	AKT/PI3K/MTOR	Aurora	BET	DNA synthesis	HDAC	HSP90	L/CH	MitoStress	Na+/K+ ATPase	Protein synthesis	PYR synthesis	Tubulin	Uncoupling
Deferasirox	10	17	36	0	83	0	0	0	52	0	0	63	0	0
Deferasirox	30	52	17	0	96	0	0	0	28	0	0	73	0	0
Deferasirox	50	46	26	0	95	0	0	0	25	0	0	74	0	0

Cluster biosimilarities

D

Cpd	Conc [µM]	AKT/PI3K/MTOR	Aurora	BET	DNA synthesis	HDAC	HSP90	L/CH	MitoStress	Na+/K+ ATPase	Protein synthesis	PYR synthesis	Tubulin	Uncoupling
PAC-1	2	15	22	0	70	0	23	1	63	0	0	41	2	0
PAC-1	6	38	30	0	92	0	0	0	41	0	0	75	0	0
PAC-1	10	44	26	0	94	0	0	0	32	0	0	80	0	0
PAC-1	30	41	17	0	96	0	0	0	48	0	0	65	0	0

Cluster biosimilarities

E

Cpd	Conc [µM]	AKT/PI3K/MTOR	Aurora	BET	DNA synthesis	HDAC	HSP90	L/CH	MitoStress	Na+/K+ ATPase	Protein synthesis	PYR synthesis	Tubulin	Uncoupling
SC144	2	41	9	0	93	0	16	0	91	0	0	59	0	0
GSK-J4	2	18	1	0	49	0	28	20	98	0	7	20	12	0
GSK-J4	10	15	0	2	44	0	26	27	98	0	11	9	22	0
JIB-04	2	46	4	0	78	0	24	0	86	0	0	54	0	0
Sanguinarine	1	11	0	7	26	4	28	27	97	6	25	0	21	0
Chelerythrine	30	6	0	8	15	4	27	29	96	5	13	0	23	0
Chelerythrine	50	0	3	8	15	2	29	26	94	3	14	0	22	0
ALK5i **1**	0.2	0	0	11	0	7	30	26	81	10	17	0	25	0
ALK5i **1**	2	3	1	9	12	4	28	29	96	6	13	0	24	0
Pyrivinium	1	4	10	0	55	0	16	16	92	8	12	14	15	0

Cluster biosimilarities

F

Cpd	Conc [µM]	AKT/PI3K/MTOR	Aurora	BET	DNA synthesis	HDAC	HSP90	L/CH	MitoStress	Na+/K+ ATPase	Protein synthesis	PYR synthesis	Tubulin	Uncoupling
Sal003	3	17	0	23	0	26	56	17	10	11	30	0	30	31
Sal003	6	33	0	28	0	18	54	39	61	1	19	0	44	44
Sal003	10	4	0	33	0	31	36	48	93	20	35	0	47	19

Cluster biosimilarities

G

Cpd	BioSim [%]	Ind [%]	Cell count [%]	Conc [µM]
Sal003	–	6	88	3
Sal003	49	23	83	6
Sal003	26	63	89	10

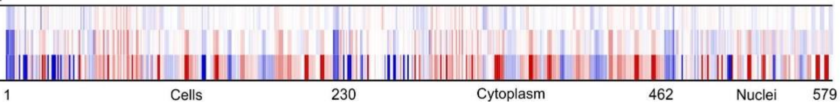

Figure S11 (related to Figure 7): Cluster biosimilarities for DFO, phenanthroline, deferasirox, PAC-1 and Sal003. (A-F) Cluster biosimilarity heatmap for the profiles of DFO (A), phenanthroline (B), deferasirox (C), PAC-1 (D), compounds biosimilar to the ciclopirox profile at 30 µM (E) and for the profiles of Sal003 (F). (G) Comparison of the profiles of Sal003. The top line profile is set as a reference profile (100 % biological similarity, BioSim) to which the following profiles are compared. Blue color: decreased feature; red color: increased feature. Cpd: compound; BioSim: biosimilarity; Ind: induction; Conc: concentration. L/CH: Lysosomotropism/cholesterol homeostasis, PYR: pyrimidine.

A

Cpd	Conc [µM]	AKT/PI3K/MTOR	Aurora	BET	DNA synthesis	HDAC	HSP90	L/CH	MitoStress	Na^+/K^+ ATPase	Protein synthesis	PYR synthesis	Tubulin	Uncoupling
		**Cluster biosimilarities**												
Rotenone	0.3	0	0	70	0	65	11	61	2	59	54	0	89	54
Rotenone	1	0	2	77	0	76	29	61	7	61	57	0	94	53
Albendazole	0.5	0	0	71	0	67	22	57	29	55	63	0	93	51
Albendazole	1	0	0	79	0	76	24	63	16	62	68	0	95	59
Mebendazole	0.6	0	10	72	0	72	27	70	10	55	56	0	90	49
Mebendazole	0.2	0	33	44	0	44	17	39	5	26	32	0	73	28
Fenbendazole	30	0	12	75	0	81	36	64	0	53	69	0	82	49
Colchicine	0.03	0	30	19	0	44	58	12	0	33	33	0	38	13
Colchicine	0.1	0	14	52	0	64	48	51	0	36	61	0	61	49
Nocodazole	0.1	0	0	76	0	75	25	62	7	58	67	0	94	54
Digoxin	1	35	19	0	40	0	0	0	0	26	2	54	0	0
Dihydroouabain	1	0	32	0	0	0	0	0	0	22	0	31	0	0
Dihydroouabain	3	25	2	0	14	0	0	0	0	35	4	23	0	7
Dihydroouabain	10	0	45	0	0	9	0	0	0	62	16	0	0	0
Dihydroouabain	30	0	6	27	0	43	0	0	0	87	25	0	22	27
Oubain	10	0	0	72	0	77	0	42	0	88	55	0	74	46
Lovastatin	10	0	0	76	0	69	19	79	34	41	43	0	69	50
Raloxifene	3	0	0	57	0	67	25	73	42	26	53	0	60	21
Raloxifene	10	0	0	75	0	76	25	89	60	34	46	0	73	49
Prazosin	10	0	58	0	4	0	27	10	10	0	0	0	35	0

B

Cpd	Conc [µM]	AKT/PI3K/MTOR	Aurora	BET	DNA synthesis	HDAC	HSP90	L/CH	MitoStress	Na^+/K^+ ATPase	Protein synthesis	PYR synthesis	Tubulin	Uncoupling
		**Cluster biosimilarities**												
Enclomiphene	1	0	0	57	0	60	34	71	59	15	44	0	58	39
Enclomiphene	3	0	0	76	0	75	11	95	43	34	56	0	68	44
Enclomiphene	10	0	0	84	0	81	12	89	35	42	69	0	78	61
Enclomiphene	30	0	0	81	0	77	16	80	0	39	69	0	78	60
Amiodarone	10	0	0	76	0	72	12	91	50	27	59	0	68	52
Amiodarone	30	0	0	76	0	73	21	87	59	34	61	0	74	52
Clozapine	10	0	0	76	0	70	6	79	7	43	33	0	65	52
Clozapine	30	0	0	77	0	77	28	92	46	33	52	0	72	48

Figure S12 (related to Figure 7): Cluster biosimilarities for the profiles of compounds studied for mitotoxicity. (A) Compounds from Seal *et al*. [3] (B) Compounds from Trapotsi *et al*.[4] Percent values are given. Cpd: compound; Conc: concentration; PYR: pyrimidine.

A

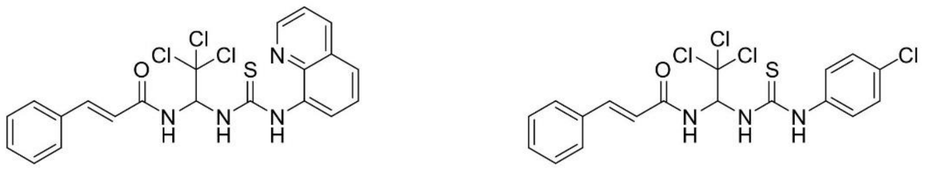

Salubrinal Sal003

B

Compound	BioSim [%]	Ind [%]	Cell count [%]	Conc [µM]
Salubrinal	–	7	98	10
	68	15	97	30
	36	22	84	50

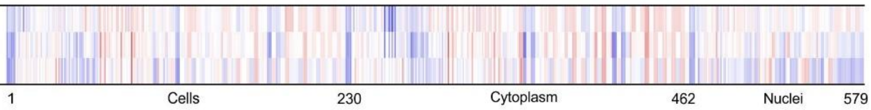

C

Compound	BioSim [%]	Ind [%]	Cell count [%]	Conc [µM]
Sal003	–	6	88	3
	49	23	83	6
	26	63	89	10

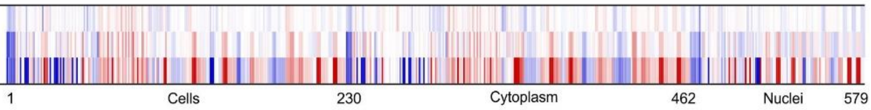

D

Cpd	Conc [µM]	AKT/PI3K/MTOR	Aurora	BET	DNA synthesis	HDAC	HSP90	L/CH	MitoStress	Na$^+$/K$^+$ ATPase	Protein synthesis	PYR synthesis	Tubulin	Uncoupling
		**Cluster biosimilarities**												
Salubrinal	10	0	0	58	0	61	36	60	28	26	55	0	64	53
	30	0	0	66	0	71	49	56	0	41	54	0	63	71
	50	25	0	38	0	55	41	34	0	33	29	0	40	64

E

		Salubrinal 10 µM	Salubrinal 30 µM	Salubrinal 50 µM	Sal003 3 µM	Sal003 6 µM
Salubrinal	30 µM	68				
	50 µM	36	77			
Sal003	3 µM	40	51	48		
	6 µM	53	31	21	49	
	10 µM	46	20	0	26	62

Figure S13 (related to Figure 7): Profile analysis for salubrinal. (A) Chemical structures of salubrinal and Sal003. (B, C) Profile similarity for salubrinal and Sal003, respectively. The top line of the heatmap profile is set as a reference profile (100 % biological similarity) to which the following profiles are compared. Blue color, decreased feature; red color, increased feature. (D) Cluster biosimilarity heatmap for salubrinal. Percent values are given. (E) Profile cross-similarity for salubrinal and Sal003. Values are biosimilarity in %. Cpd: compound; BioSim: biosimilarity; Ind: induction; Conc: concentration. L/CH: Lysosmotropism/cholesterol homeostasis; PYR: pyrimidine.

A

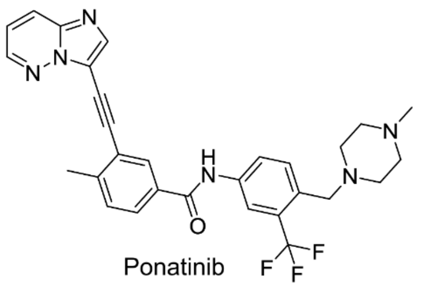

B

Cluster biosimilarities for ponatinib

Conc [µM]	AKT/PI3K/MTOR	Aurora	BET	DNA synthesis	HDAC	HSP90	L/CH	MitoStress	Na+/K+ ATPase	Protein synthesis	PYR synthesis	Tubulin	Uncoupling
1	0	0	71	0	71	35	74	40	33	62	0	73	45
2	0	0	83	0	80	28	93	36	46	51	0	82	59

C

Compound	BioSim [%]	Ind [%]	Cell count [%]	Conc [µM]
SB525334	–	45	97	30
Ponatinib	78	69	73	1
Ponatinib	82	65	63	2

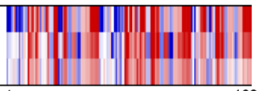

Figure S14 (related to Figure 7): Profile analysis for ponatinib. (A) Chemical structure of ponatinib. (B) Cluster biosimilarity heatmap for ponatinib. Percent values are given. (C) Profile similarity for ponatinib compared to SB525334. The top line of the heatmap profile is set as a reference profile (100 % biological similarity) to which the following profiles are compared. Blue color, decreased feature; red color, increased feature. Only the MitoTracker-related features of the MitoStress cluster subprofile were used for the comparison. Cpd: compound; BioSim: biosimilarity; Ind: induction; Conc: concentration. L/CH: Lysosmotropism/cholesterol homeostasis; PYR: pyrimidine.

Supporting Tables

Table S1 (related to Figure 1): CPA features for cicloprox (see separate XLS file).

Table S2 (related to Figure 2): Compounds that are biosimilar in CPA to ciclopirox at 30 µM at the indicated concentrations.

Name	Conc [µM]	Induction [%]	BioSim [%]	Known activity
Ciclopirox (olamine) 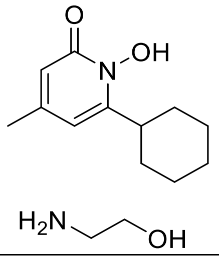	30	59.2	100	Metal ion chelator
	50	64.8	97.5	
ML228 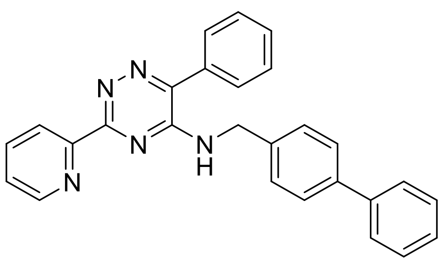	3	65.5	95.7	Hypoxia Inducible Factor (HIF) pathway activator
	10	54.4	92.7	
NSC319726 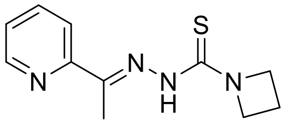	2	53.2	94.6	p53(R175) mutant reactivator
SC144 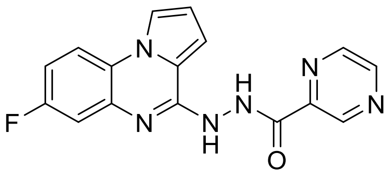	2	52.3	92.2	gp130 (IL6-beta) inhibitor [5]
GSK-J4 (hydrochloride) 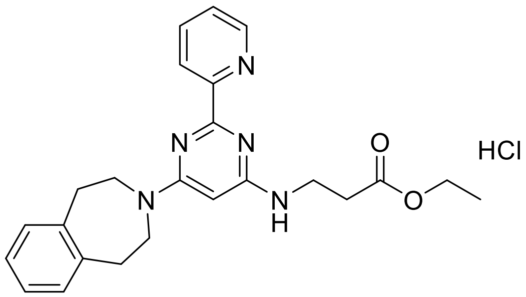	10	46.3	86.5	Dual inhibitor of H3K27me3/me2-demethylases JMJD3/KDM6B and UTX/KDM6A
	2	40.6	83.5	

Name	Conc [µM]	Induction [%]	BioSim [%]	Known activity
BLU9931 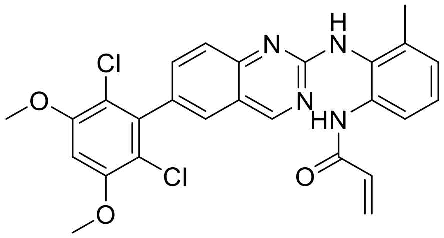	10	51.5	84.2	Fibroblast growth factor receptor 4 (FGFR4) inhibitor
Dephostatin 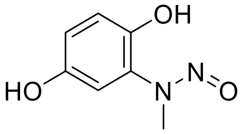	30	37	81.4	CD45 protein tyrosine kinase inhibitor.
JIB-04 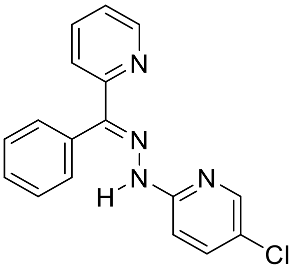	2	44.9	81.1	Pan-selective Jumonji histone demethylase inihibitor
Sanguinarine chloride 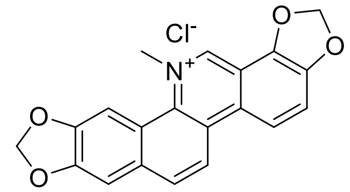	1	36.3	78.6	Inhibitor of Mg^{2+} and Na^{+} /K^{+} ATPase [6]
Chelerythrine (chloride) 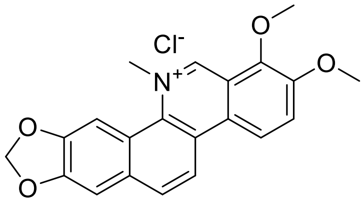	30	33.2	78.1	Inhibitor of protein kinase C [7]
	50	31.4	77.3	
ALK5i **1** 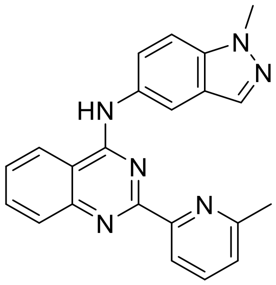	2	30.4	77.8	Inhibitor of TGF-beta receptor type I (ALK5) [8]

Name	Conc [µM]	Induction [%]	BioSim [%]	Known activity
Pyrvinium pamoate 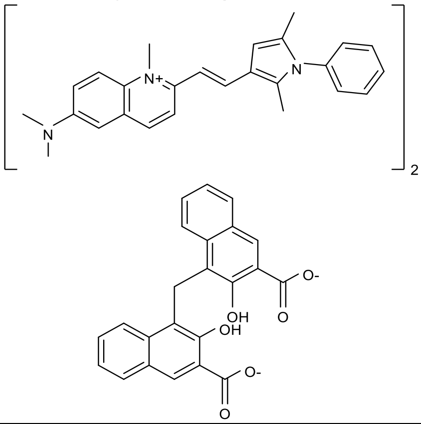	1	44.4	76.9	Metabolic inhibitor [9]

Table S3 (related to Figure 2). Compounds that are biosimilar in CPA to ciclopirox at 30 µM at the indicated concentrations when only MitoTracker-related features are considered. Conc: concentration; BioSim: biosimilarity

Trivial Name	Conc. [µM]	Induction [%]	BioSim [%]	Known activity
Ciclopirox (olamine)	30.0	59.2	100.0	metal ion chelator
Ciclopirox (olamine)	50.0	64.8	99.4	metal ion chelator
GSK-J4 (hydrochloride)	10.0	46.3	97.3	dual inhibitor of H3K27me3/me2-demethylases JMJD3/KDM6B and UTX/KDM6A
ML228	3.0	65.5	97.0	Hypoxia Inducible Factor (HIF) pathway activator
Chelerythrine (chloride)	30.0	33.2	96.7	Inhibitor of protein kinase C
NSC319726	2.0	53.2	95.9	p53(R175) mutant reactivator
SB 525334	50.0	45.9	95.8	Transforming growth factor b1 receptor (ALK5) inhibitor
Sal003	10.0	62.9	95.6	Inhibitor of the eukaryotic translation initiation factor 2a (eIF2a) phosphatase
Chelerythrine (chloride)	50.0	31.4	95.5	Inhibitor of protein kinase C
ML228	10.0	54.4	95.2	Hypoxia Inducible Factor (HIF) pathway activator
Berberine (chloride hydrate)	30.0	25.4	94.6	Induces reactive oxygen species (ROS) generation and inhibits DNA topoisomerase
Berberine (chloride hydrate)	50.0	31.8	94.5	Induces reactive oxygen species (ROS) generation and inhibits DNA topoisomerase
SB 525334	30.0	44.7	93.4	Transforming growth factor b1 receptor (ALK5) inhibitor
SC144	2.0	52.3	93.2	gp130 (IL6-beta) inhibitor
Calcimycin, A23187, Calcium ionophore A23187	10.0	60.3	92.9	Ca^2+ ionophore
Dequalinium dichloride	10.0	55.4	92.3	Blocker of apamin-sensitive K+ channels
Oligomycin A	10.0	34.0	89.2	Mitochondrial F_0F_1-ATPase inhibitor
BLU9931	10.0	51.5	89.0	Fibroblast growth factor receptor 4 (FGFR4) inhibitor
Chelerythrine (chloride)	2.0	17.8	88.5	Inhibitor of protein kinase C
JIB-04	2.0	44.9	87.1	Pan-selective Jumonji histone demethylase inihibitor
Calcimycin, A23187, Calcium ionophore A23187	0.3	61.1	87.1	Ca^{2+} ionophore
Phenserine	30.0	63.6	86.7	Non-competitive acetylcholinesterase (AChE) inhibitor.
Halofantrine hydrochloride	3.0	23.8	85.4	Blocker of delayed rectifier potassium current via the inhibition of human-ether-a-go-go-related gene (HERG) channel

Table S4 (related to Figure 5): Proteins regulated by CPX 10 and 30 µM (see separate XLS file).

Table S5 (related to Figure 5 and 6): Proteome profiling data (see separate XLS file).

Table S6 (related to Figure 5 and 6): Ingenuity Pathways Analysis analysis (see separate XLS file).

Table S7 (related to Figure 5 and 6): Overlap of downregulated proteins in Quiros et al. [10] and this study. The table lists proteins, which were reported by Quiros et al. to be downregulated upon the treatment with mitochondrial stressor, that were also downregulated by ciclopirox (CPX), GSK-J4, SB525334 or compound **2** (cpd **2**). Data for SB525334 is shown for log fold change (FC) of 0.3. and 0.2 as only little changes were detected at log FC of 0.2.

30 µM CPX	GSK-J4	SB525334 log FC 0.3	SB525334 log FC 0.2	Cpd 2
ADCK4		METTL17	ADCK4	DAP3
ATF7IP			FDFT1	FADS2
CYP51A1			METTL17	FDFT1
DAB2			MRPL24	MRPS12
DAP3			MRPL28	MRPS7
ERAL1			MRPL41	
FADS2			NDUFA2	
FDFT1			NDUFA5	
ICT1			RAL1	
MRPL11				
MRPL17				
MRPL18				
MRPL19				
MRPL24				
MRPL28				
MRPL3				
MRPL30				
MRPL40				
MRPL42				
MRPL43				
MRPL45				
MRPL51				
MRPS15				
MRPS18A				
MRPS18B				
MRPS2				
MRPS22				
MRPS23				
MRPS28				
MRPS35				
MRPS5				
MRPS7				
MRPS9				
MTG1				
NDUFA2				
NDUFA5				
NDUFA6				
TMEM50A				

Table S8 (related to Figure 5 and 6): Upregulated genes and proteins in Quiros et al. [10] and this study. The table lists genes and proteins, which were reported by Quiros et al. to be downregulated upon the treatment with mitochondrial stressor, that were also upregulated by ciclopirox (CPX), GSK-J4, SB525334 or compound **2** (cpd **2**). Data for SB525334 is shown for log fold change (FC) of 0.3. and 0.2 as only little changes were detected at log FC of 0.2.

30 µM CPX	GSK-J4	SB525334 log FC 0.3	SB525334 log FC 0.2	Cpd 2
DNASE2<br>GRB10<br>SARS<br>VLDLR	SLC7A11<br>VLDLR		DDR2<br>PSAT1	AMIGO2<br>ASNS<br>CTH<br>DDR2<br>PCK2<br>PSAT1<br>PSPH<br>SESN2<br>SLC1A4<br>SLC1A5<br>SLC7A11<br>VLDLR

Table S9 (related to Figure 7): MitoStress cluster defining compounds.

Trivial_Name	Induction [%]	Conc [µM]	Cell count [%]	MitoStress cluster similarity
Ciclopirox (olamine)	59	30	75	93
Ciclopirox (olamine)	65	50	72	96
ML228	66	3	71	94
ML228	54	10	65	95
SC144	52	2	69	91
GSK-J4 (hydrochloride)	41	2	85	98
GSK-J4(hydrochloride)	46	10	80	98
JIB-04	45	2	79	86
Sanguinarine (chloride)	36	1	71	96
Chelerythrine (chloride)	33	30	86	96
Chelerythrine(chloride)	31	50	79	94
Compound **1**	30	2	92	96
Pyrvinium pamoate	44	1	69	92
Dephostatin	37	30	75	86
BLU9931	52	10	70	92
NSC319726	53	2	69	93

Supporting Movies

Supplementary Movie S1. U-2OS cells treated with 0.5 % DMSO as a control.

Supplementary Movie S2. U-2OS cells treated with 30 μM ciclopirox for 24 h.

Supplementary Movie S3. U-2OS cells treated with 2 μM GSK-J4 for 24 h.

HPLC traces

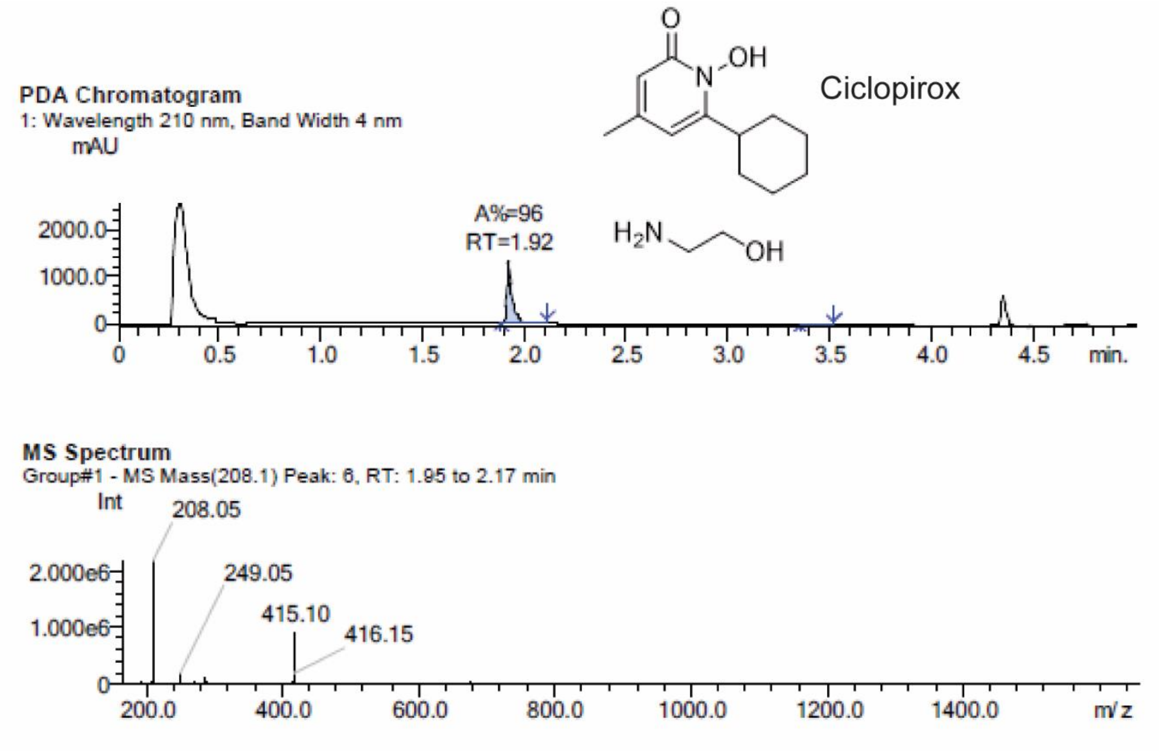

Ciclopirox
PDA Chromatogram
1: Wavelength 210 nm, Band Width 4 nm
mAU
A%=96
RT=1.92
MS Spectrum
Group#1 - MS Mass(208.1) Peak: 6, RT: 1.95 to 2.17 min
Int
208.05
249.05
415.10
416.15
m/z
min.

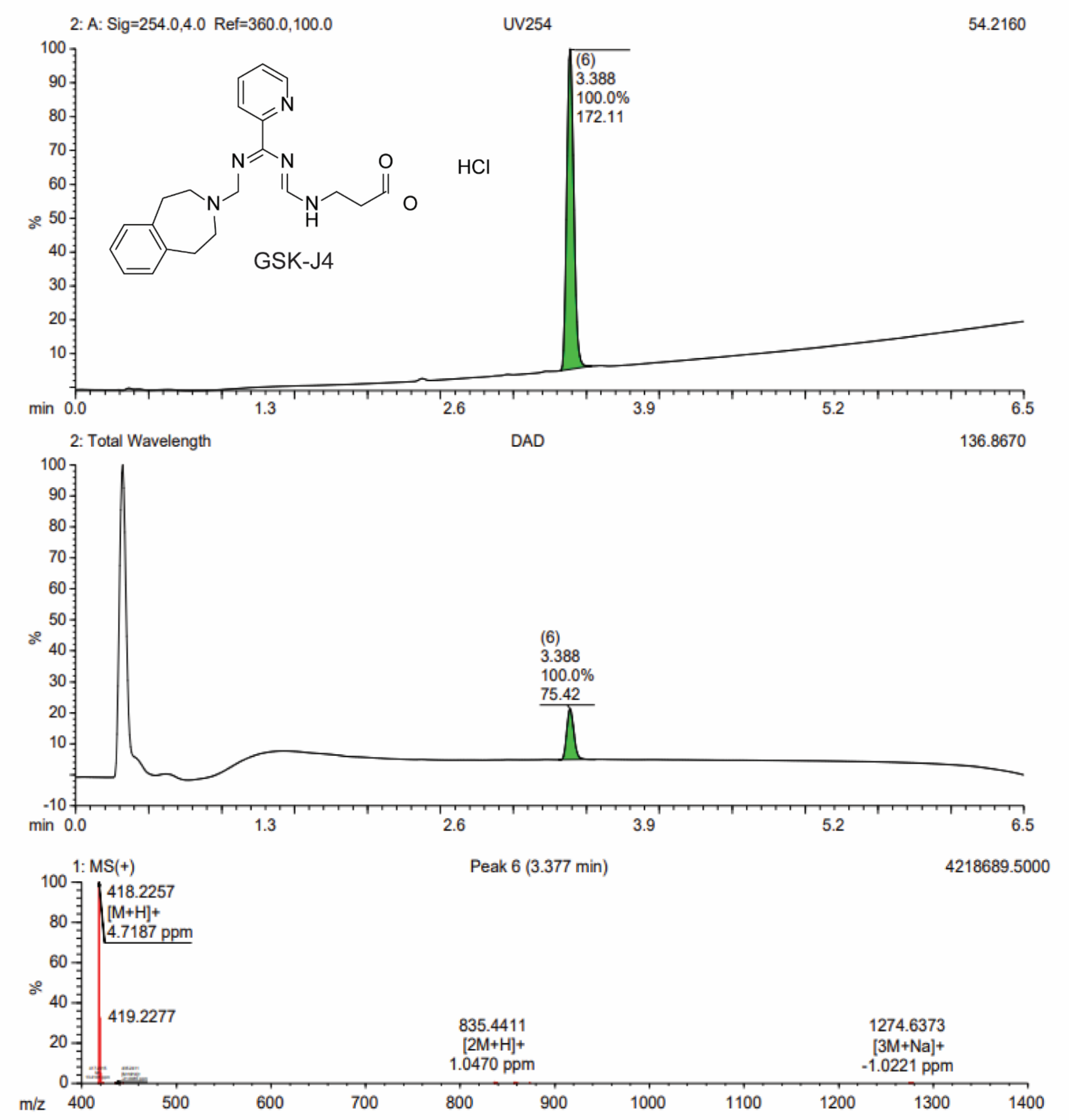
2: A: Sig=254.0,4.0 Ref=360.0,100.0
UV254
54.2160
(6)
3.388
100.0%
172.11
HCl
GSK-J4
2: Total Wavelength
DAD
136.8670
(6)
3.388
100.0%
75.42
1: MS(+)
Peak 6 (3.377 min)
4218689.5000
418.2257
[M+H]+
4.7187 ppm
419.2277
835.4411
[2M+H]+
1.0470 ppm
1274.6373
[3M+Na]+
-1.0221 ppm
m/z

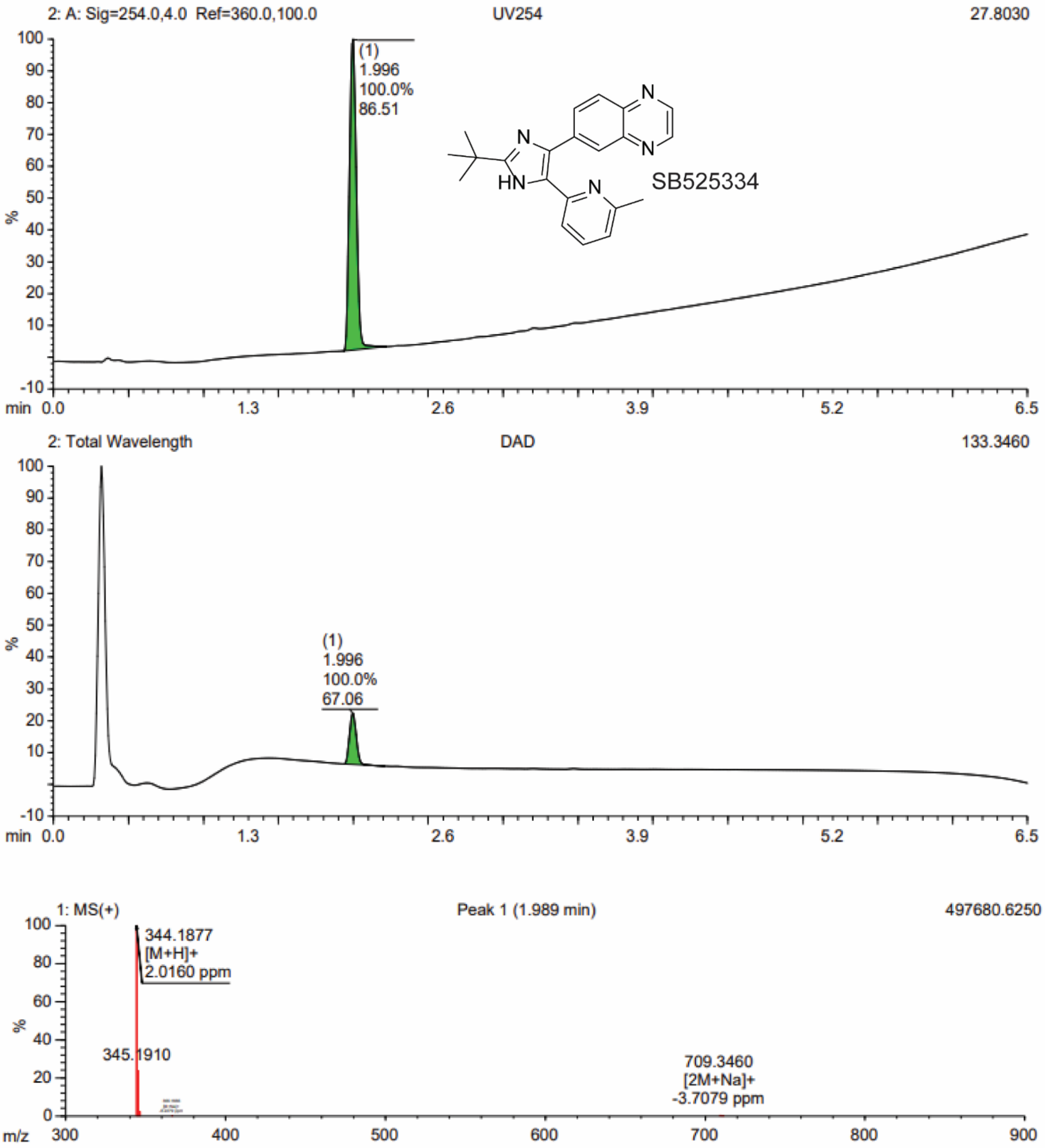
2: A: Sig=254.0,4.0 Ref=360.0,100.0
UV254
27.8030
(1)
1.996
100.0%
86.51
SB525334
N
N
N
HN
N
%
min
2: Total Wavelength
DAD
133.3460
(1)
1.996
100.0%
67.06
1: MS(+)
Peak 1 (1.989 min)
497680.6250
344.1877
[M+H]+
2.0160 ppm
345.1910
709.3460
[2M+Na]+
-3.7079 ppm
m/z

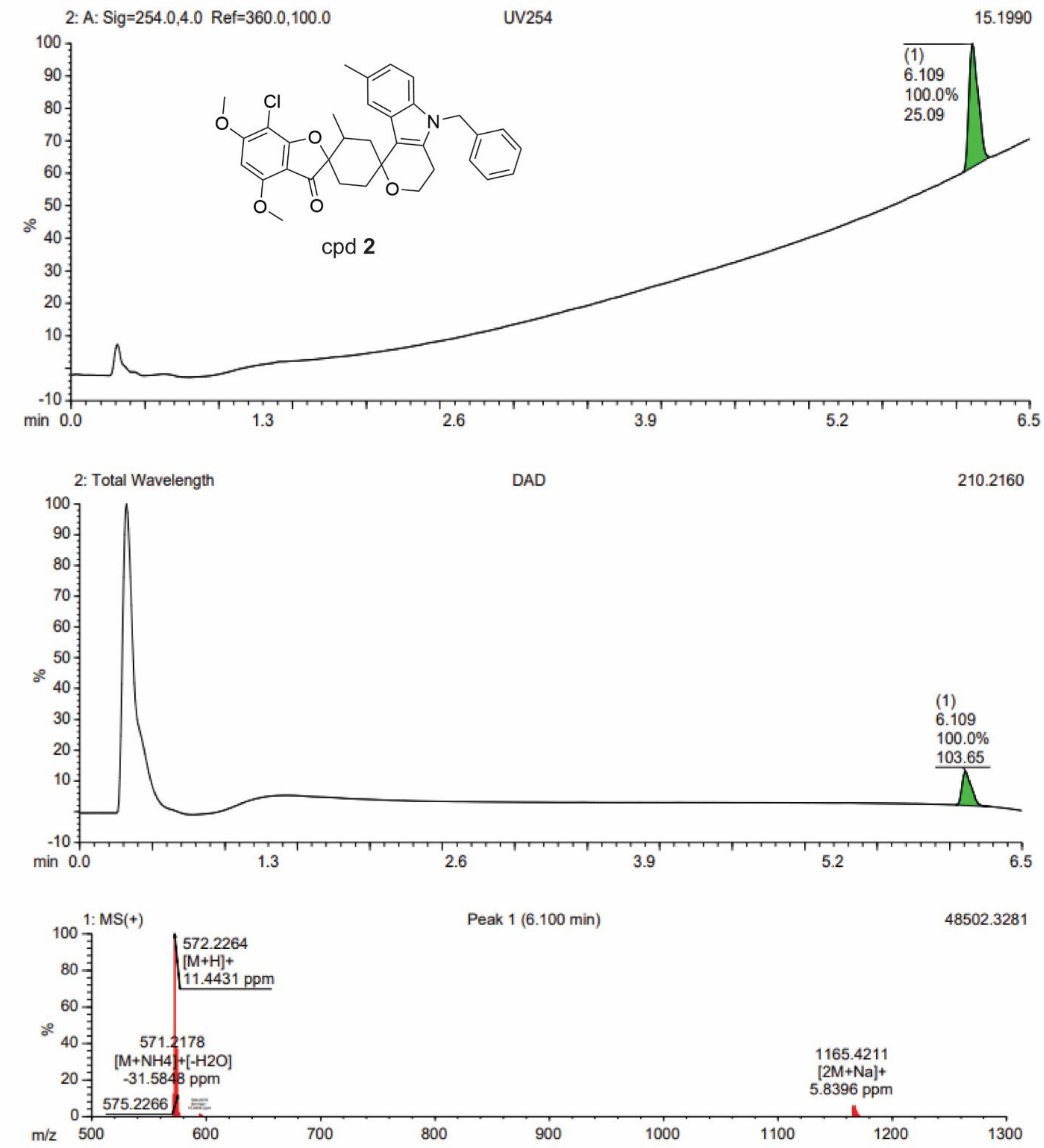
2: A: Sig=254.0,4.0 Ref=360.0,100.0
UV254
15.1990
(1)
6.109
100.0%
25.09
cpd 2
%
min
2: Total Wavelength
DAD
210.2160
(1)
6.109
100.0%
103.65
1: MS(+)
Peak 1 (6.100 min)
48502.3281
572.2264
[M+H]+
11.4431 ppm
571.2178
[M+NH4]+[-H2O]
-31.5848 ppm
575.2266
1165.4211
[2M+Na]+
5.8396 ppm
m/z

References

(1) Goedhart, J.; Luijsterburg, M. S., Volcanoser Is a Web App for Creating, Exploring, Labeling and Sharing Volcano Plots, *Sci Rep* **2020**, *10*, 20560.

(2) Pahl, A.;Scholermann, B.;Lampe, P.;Rusch, M.;Dow, M.;Hedberg, C.;Nelson, A.;Sievers, S.;Waldmann, H.; Ziegler, S., Morphological Subprofile Analysis for Bioactivity Annotation of Small Molecules, *Cell Chem Biol* **2023**, *30*, 839-853 e837.

(3) Seal, S.;Carreras-Puigvert, J.;Trapotsi, M.-A.;Yang, H.;Spjuth, O.; Bender, A., Integrating Cell Morphology with Gene Expression and Chemical Structure to Aid Mitochondrial Toxicity Detection, *Communications Biology* **2022**, *5*, 858.

(4) Trapotsi, M.-A.;Mouchet, E.;Williams, G.;Monteverde, T.;Juhani, K.;Turkki, R.;Miljkovic, F.;Martinsson, A.;Mervin, L.; Pryde, K. R., Cell Morphological Profiling Enables High-Throughput Screening for Proteolysis Targeting Chimera (Protac) Phenotypic Signature, *ACS Chemical Biology* **2022**, *17*, 1733-1744.

(5) Lu, T.;Tang, J.;Shrestha, B.;Heath, B. R.;Hong, L.;Lei, Y. L.;Ljungman, M.; Neamati, N., Up-Regulation of Hypoxia-Inducible Factor Antisense as a Novel Approach to Treat Ovarian Cancer, *Theranostics* **2020**, *10*, 6959.

(6) Croaker, A.;King, G. J.;Pyne, J. H.;Anoopkumar-Dukie, S.; Liu, L., Sanguinaria Canadensis: Traditional Medicine, Phytochemical Composition, Biological Activities and Current Uses, *International journal of molecular sciences* **2016**, *17*, 1414.

(7) Chen, N.;Qi, Y.;Ma, X.;Xiao, X.;Liu, Q.;Xia, T.;Xiang, J.;Zeng, J.; Tang, J., Rediscovery of Traditional Plant Medicine: An Underestimated Anticancer Drug of Chelerythrine, *Front Pharmacol* **2022**, *13*, 906301.

(8) Gellibert, F.;Fouchet, M.-H.;Nguyen, V.-L.;Wang, R.;Krysa, G.;de Gouville, A.-C.;Huet, S.; Dodic, N., Design of Novel Quinazoline Derivatives and Related Analogues as Potent and Selective Alk5 Inhibitors, *Bioorganic & medicinal chemistry letters* **2009**, *19*, 2277-2281.

(9) Ishii, I.;Harada, Y.; Kasahara, T., Reprofiling a Classical Anthelmintic, Pyrvinium Pamoate, as an Anti-Cancer Drug Targeting Mitochondrial Respiration, *Frontiers in oncology* **2012**, *2*, 137.

(10) Quirós, P. M.;Prado, M. A.;Zamboni, N.;D'Amico, D.;Williams, R. W.;Finley, D.;Gygi, S. P.; Auwerx, J., Multi-Omics Analysis Identifies Atf4 as a Key Regulator of the Mitochondrial Stress Response in Mammals, *Journal of Cell Biology* **2017**, *216*, 2027-2045.
